# Measuring Collaboration Through Concurrent Electronic Health Record Usage: Network Analysis Study

**DOI:** 10.2196/28998

**Published:** 2021-09-03

**Authors:** Patrick Li, Bob Chen, Evan Rhodes, Jason Slagle, Mhd Wael Alrifai, Daniel France, You Chen

**Affiliations:** 1 Department of Computer and Information Science University of Pennsylvania Philadelphia, PA United States; 2 Epithelial Biology Center Vanderbilt University Medical Center Nashville, TN United States; 3 Department of Anesthesiology Vanderbilt University Medical Center Nashville, TN United States; 4 Department of Pediatric Vanderbilt University Medical Center Nashville, TN United States; 5 Department of Biomedical Informatics Vanderbilt University Medical Center Nashville, TN United States; 6 Department of Computer Science Vanderbilt University Nashville, TN United States

**Keywords:** collaboration, electronic health records, audit logs, health care workers, neonatal intensive care unit, network analysis, clustering, visualization, concurrent interaction, human-computer interaction, survey instrument, informatics framework, secondary data analysis

## Abstract

**Background:**

Collaboration is vital within health care institutions, and it allows for the effective use of collective health care worker (HCW) expertise. Human-computer interactions involving electronic health records (EHRs) have become pervasive and act as an avenue for quantifying these collaborations using statistical and network analysis methods.

**Objective:**

We aimed to measure HCW collaboration and its characteristics by analyzing concurrent EHR usage.

**Methods:**

By extracting concurrent EHR usage events from audit log data, we defined concurrent sessions. For each HCW, we established a metric called concurrent intensity, which was the proportion of EHR activities in concurrent sessions over all EHR activities. Statistical models were used to test the differences in the concurrent intensity between HCWs. For each patient visit, starting from admission to discharge, we measured concurrent EHR usage across all HCWs, which we called temporal patterns. Again, we applied statistical models to test the differences in temporal patterns of the admission, discharge, and intermediate days of hospital stay between weekdays and weekends. Network analysis was leveraged to measure collaborative relationships among HCWs. We surveyed experts to determine if they could distinguish collaborative relationships between high and low likelihood categories derived from concurrent EHR usage. Clustering was used to aggregate concurrent activities to describe concurrent sessions. We gathered 4 months of EHR audit log data from a large academic medical center’s neonatal intensive care unit (NICU) to validate the effectiveness of our framework.

**Results:**

There was a significant difference (*P*<.001) in the concurrent intensity (proportion of concurrent activities: ranging from mean 0.07, 95% CI 0.06-0.08, to mean 0.36, 95% CI 0.18-0.54; proportion of time spent on concurrent activities: ranging from mean 0.32, 95% CI 0.20-0.44, to mean 0.76, 95% CI 0.51-1.00) between the top 13 HCW specialties who had the largest amount of time spent in EHRs. Temporal patterns between weekday and weekend periods were significantly different on admission (number of concurrent intervals per hour: 11.60 vs 0.54; *P*<.001) and discharge days (4.72 vs 1.54; *P*<.001), but not during intermediate days of hospital stay. Neonatal nurses, fellows, frontline providers, neonatologists, consultants, respiratory therapists, and ancillary and support staff had collaborative relationships. NICU professionals could distinguish high likelihood collaborative relationships from low ones at significant rates (3.54, 95% CI 3.31-4.37 vs 2.64, 95% CI 2.46-3.29; *P*<.001). We identified 50 clusters of concurrent activities. Over 87% of concurrent sessions could be described by a single cluster, with the remaining 13% of sessions comprising multiple clusters.

**Conclusions:**

Leveraging concurrent EHR usage workflow through audit logs to analyze HCW collaboration may improve our understanding of collaborative patient care. HCW collaboration using EHRs could potentially influence the quality of patient care, discharge timeliness, and clinician workload, stress, or burnout.

## Introduction

The measurement of coordinated collaboration in health care systems has proven to be important for providing better quality care [1-9]. Numerous studies have correlated collaboration with quality of care [1-3], patient safety [4-6], and clinical outcomes [7,8]. No universal guidelines exist to study collaboration in health care organizations (HCOs). Existing studies approached collaboration by relying on surveys, written reports, and interviews as a basis for gauging collaboration [1-9]. Further, they examined communication, teamwork, and problem-solving in HCOs, noting that interprofessional team functions are often suboptimal [3,7]. In addition, these studies identified barriers to successful interprofessional collaboration, including power dynamics, poor communication patterns, and incomplete understanding of roles and responsibilities [1-9]. However, existing studies seldom examine collaborative activities in the context of electronic health record (EHR) system usage. EHR systems provide a virtual environment for a diverse collection of health care workers (HCWs) to exchange accurate, detailed, and timely information electronically [10-12].

As EHRs have grown in adoption, the proportion of collaboration among HCWs involving EHR systems has increased as well [13-15]. For instance, a respiratory therapist noted, in an EHR, that a patient had an increased need for oxygen. At the same time, a nurse documented the same patient’s vital signs and noted the presentation of tachypnea. Next, an attending physician reviewed the vitals and respiratory rate, and prescribed the patient a diuretic [16]. Here, three HCWs experienced latent (inexplicit) collaboration through the EHR system that may not have been flagged by HCOs. HCWs may spend a considerable amount of time in latent collaborations in caring for patients through EHR systems [17-19]. The relationships among latent collaborations, care quality, and patient safety, however, have been understudied due to a lack of metrics or concepts describing collaboration of this nature.

Highly granular and widely available EHR audit logs document HCW activities occurring within EHRs [20-23] and can be used to model latent collaboration among HCWs and the respective interactions between HCWs and EHR systems [13,16,24-29]. Typically, each event documented in an audit log includes a timestamp, the type of action involved, the involved HCW and patient IDs, and further metadata, such as HCW specialties, patient demographics, and health conditions [13,16,20-29]. EHR audit logs have been widely used to measure health care organizational structures [20,25], clinical workflows [20,30,31], trauma care team structures [13,20,26], and intensive care unit care structures [16,27-29]. Existing studies have investigated audit log data at a coarse-grained level to build connections between HCWs [13,16,20-31], and thus, much of the contextual information (eg, HCW-EHR system interactions) is lost. For instance, coarse-grained latent interactions between HCWs have previously been defined by shared interactions with the same patients on the same day or during the same patient encounter [26-29]. We demonstrate that audit logs enable the study of latent collaborative activities at a highly granular level.

In this study, we propose a robust framework for the investigation of latent collaboration through concurrent EHR utilization. Using this framework, we describe a case study showcasing its usage in the neonatal intensive care unit (NICU) of a large academic medical center consisting of neonatologists, neonatal fellows, neonatal frontline providers, neonatal nurses, respiratory therapists, consultants, ancillary staff, and support staff. In the NICU, the density of audit logs per patient episode is very high, and it is an ideal environment for investigating latent collaboration [16,28].

## Methods

### Overview

**Figure 1:**
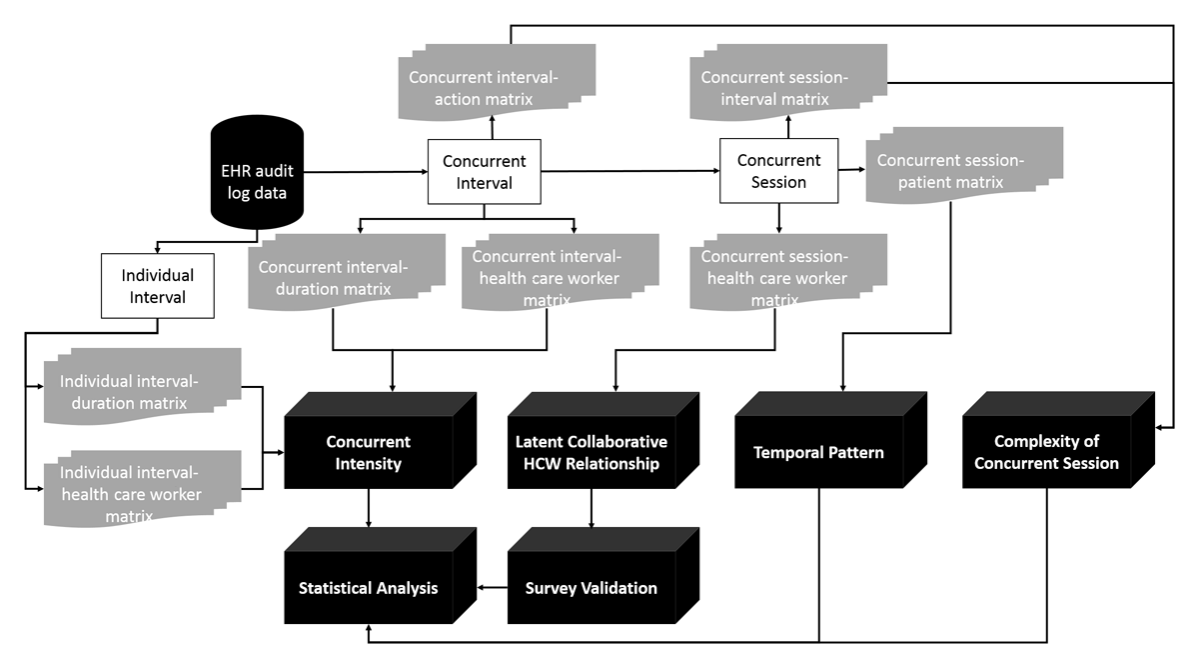
A workflow diagram showing our framework on learning concurrent intensity, latent collaborative HCW relationships, temporal trends of concurrent EHR usage, and concurrent session complexity from EHR audit log data. EHR: electronic health record; HCW: health care worker.

In this section, we describe how we defined and calculated individual intervals, concurrent intervals, and concurrent sessions from the audit log data. We defined the core components of our proposed framework for measuring latent collaboration and its characteristics, via audit log data, which involve concurrent intensity (proportion of concurrent intervals and time spent on those intervals), latent collaborative HCW relationships, temporal patterns (weekday vs weekend or admission vs discharge temporal trends of concurrent EHR usage), and the complexity of concurrent sessions. Figure 1 shows the workflow of learning latent collaboration and its characteristics from the audit log data.

### Events in EHR Audit Logs

An event is a single row of an audit log entry containing the HCW ID, patient ID, action ID, and time stamp. Thus, an event describes an action that an HCW performed on an EHR of a patient at a specific time. The action ID corresponds to the type of action performed, such as typing a progress note, accessing patient demographics, refilling medications, reviewing cholesterol test results, and so on. Table 1 shows a list of events performed by two HCWs (anonymized IDs A and B) on EHRs of two patients (anonymized IDs 1 and 2). These events are retrieved from EPIC EHR audit logs. Further definitions of the events can be found at Epic’s EHR UserWeb [32].

**Table 1:** Examples of events by health care workers.

Healthcare worker ID	Patient ID	Event action	Timestamp
A	1	FLOWSHEETS DATA SAVED	4/5/2020 2:14:25
A	1	CHART REVIEW ENCOUNTERS TAB SELECTED	4/5/2020 2:15:00
A	1	CHART REVIEW OTHER ORDERS TAB SELECTED	4/5/2020 2:18:23
A	1	HISTORY ACTIVITY ACCESSED	4/5/2020 2:19:53
A	1	FLOWSHEETS DATA COPIED FORWARD	4/5/2020 2:21:32
A	1	CHART REVIEW MEDICATIONS TAB SELECTED	4/5/2020 2:22:23
B	2	VISIT NAVIGATOR TEMPLATE LOADED	12/3/2020 06:31:27
B	2	SNAPSHOT REPORT VIEWED	12/3/2020 06:33:11
B	2	CHART REVIEW NOTES	12/3/2020 06:34:41
B	2	CHART REVIEW ENCOUNTER	12/3/2020 06:36:27
B	2	CHART REVIEW RESULTS	12/3/2020 06:37:33
B	2	CHART REVIEW OTHER ORDERS	12/3/2020 06:39:27

### Creating Intervals From Events

**Figure 2:**
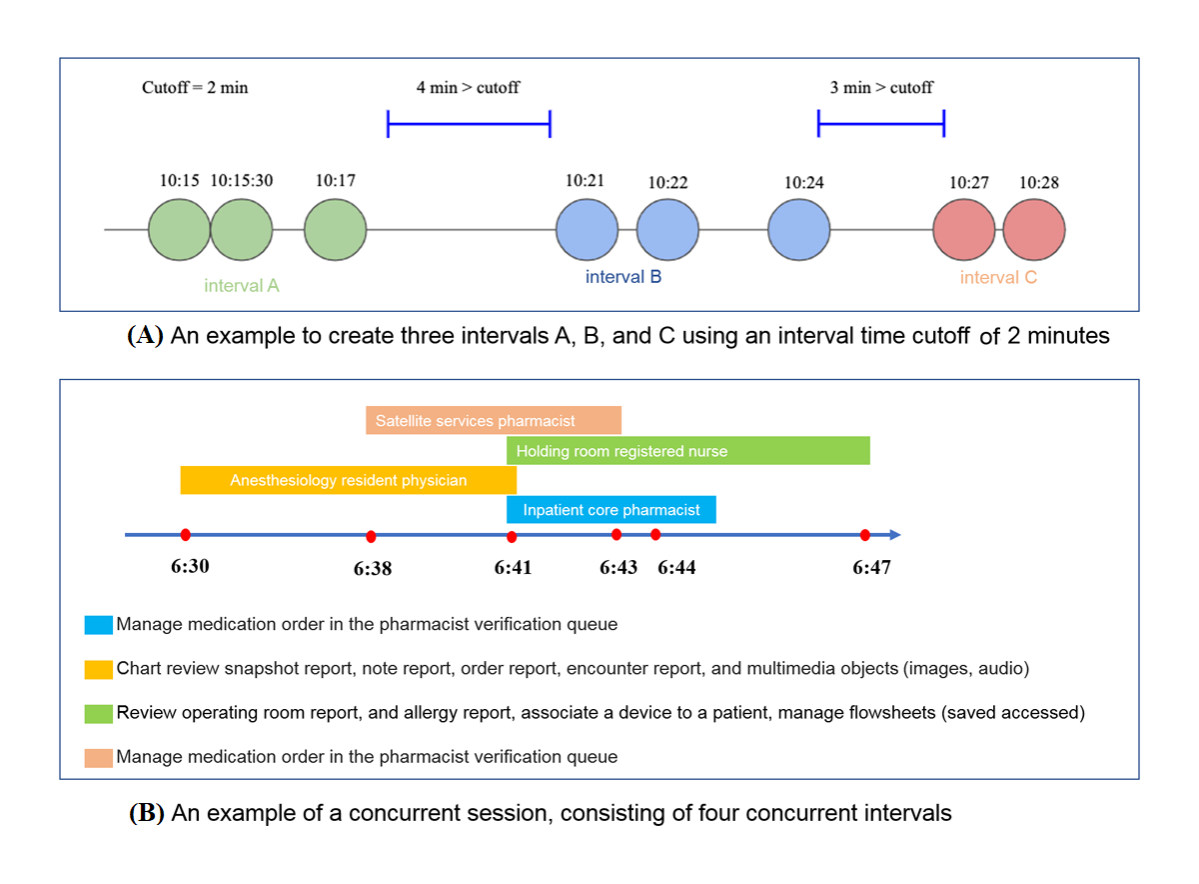
Examples of creating intervals from events (A) and defining a concurrent session based on overlapped intervals (B).

We defined an interval as an ordered list of events that occur sequentially until two events are spaced in time by more than a certain cutoff (note that these events must be from the same HCW and the same patient). Each interval has start and stop times, corresponding to the first event and last event times in the interval. Intervals also have a duration metric, which is simply the difference between the start and stop times. Figure 2A provides a more detailed example using 2 minutes as a cutoff. This interval definition aims to divide an HCW’s EHR actions into a set of segments, similar to an order session or a series of orders placed by a clinician for a single patient, defined in a previous report [33].

A “knee point” finding algorithm, described by Satopaa et al, was used to estimate the cutoff used [34]. Our previous study used such a strategy and identified clinically meaningful intervals for the sessionization of audit logs [35]. This strategy has been used before in finding the operating points of complex systems and is defined more formally, for any continuous function *f*, as follows:

K*_f_* (*x*) = *f*′′(*x*) / (1 + *f*′(*x*)^2^)^1.5^
**(1)**

K*_f_* (*x*) represents the closed form of the curvature *f* at any point as a function of its first and second derivatives. We find *x* through the Kneedle algorithm, which maximizes this curvature [34].

### Creating Concurrent Sessions From Intervals

We defined a concurrent session as a set of temporally overlapping intervals performed by different HCWs on EHRs of the same patient. We assumed that concurrent sessions can indicate who works with whom given that they are simultaneously performing EHR actions to manage a single patient. A concurrent interval is any interval that is part of a concurrent session; likewise, an individual interval is any interval that is not a part of any concurrent session. Concurrent intervals of a session have overlaps that are greater than zero. Figure 2B provides a more detailed example of a concurrent session made up of four concurrent intervals.

### Workday Definition

We found that sometimes HCWs spend only a small amount of time (eg, 5 minutes per 24 hours) interacting with the EHRs of patients. We denoted these lower activity days as inactive EHR workdays and assumed that such workdays have little impact on measuring latent collaboration and its characteristics. Thus, we only investigated active EHR days in this study. An active EHR day was defined as a day (24 hours) where the sum of all the HCW interval durations in that day exceeds a certain amount of time, or the workday time cutoff. This cutoff value is determined by different clinical settings (eg, NICU or primary care) and the respective HCW time spent interacting with EHRs. We relied on expert knowledge in EHR utilization to determine the workday cutoff value.

### Creating Intermediate Data Matrices

Based on the concurrent sessions, we generated eight intermediate matrices (Figure 1) describing latent collaboration and its characteristics. For instance, the associations between concurrent intervals and actions were stored in the concurrent interval-action matrix, and the associations between concurrent intervals and concurrent sessions were stored in the concurrent interval-concurrent session matrix. The intermediate data were used in the following analysis.

### Measuring the Concurrent Intensity of an HCW

Given the definitions previously discussed, we can create attributes for each HCW. These attributes include HCW specialty (eg, neonatologist and neonatal nurse), individual intervals, concurrent intervals, durations of both individual and concurrent intervals, EHR workdays, and the durations of these EHR workdays. We leveraged these attributes to measure the proportion of concurrent intervals over all recorded intervals and the proportion of time spent on concurrent intervals. These two attributes comprise the concurrent intensity of an HCW. We measured the concurrent intensity per day, excluding inactive EHR workdays. The daily concurrent intensity, along with EHR time on active EHR workdays, was used to describe the time characteristics of an HCW in EHR systems. We used Spearman rank correlation to measure the association between daily time in EHRs and daily time spent on concurrent intervals for HCWs affiliated with the same specialty attribute. This tests the null hypothesis that there is no association between daily time spent on concurrent intervals and daily time spent on EHRs across all HCWs affiliated with the same specialty. Moreover, we applied a one-way analysis of variance (ANOVA) to test the significance of differences in the concurrent intensity and EHR time on active workdays between specialties at a significance level of .05. The null hypothesis is that there are no significant differences in the concurrent intensity/EHR time between HCWs with disparate specialties. All statistical analyses, including those in the following sections, were performed using R 4.0.4 (R Foundation for Statistical Computing). The four matrices, as shown in Figure 1, were used to quantify concurrent intensity for each HCW.

### Measuring and Validating Latent Collaborative Relationships Between HCWs

The HCW-concurrent session matrix was leveraged to measure relationships between HCWs with respect to their participation in concurrent sessions. In this study, we used the number of co-affiliated sessions between pairs of HCWs to measure the relationship’s strength. Based on these weightings between HCWs, we created a network of HCWs to describe their latent collaborations. We used K-core analyses to identify a subgraph depicting core latent collaboration among HCWs in EHR systems. Each HCW within the K-core subgraph is connected to at least K other HCWs, and each respective HCW is considered as one core of the whole collaboration network. Gephi, an open-sourced network analysis and visualization tool, was used in this study [36].

We assumed that if the learned classes of the collaborative relationships (high and low strength) are consistent with the psychological expectations of HCWs, our approaches measuring latent collaborative relationships are plausible. To assess if HCWs can distinguish between likelihoods of collaborative relationships derived from EHRs, we divided putative collaborative relationships into the following two groups: high and low likelihoods. We randomly selected a set of collaborative relationships from the high and low groups, which were assessed by invited experts in an online survey. The experts who responded to the survey were asked questions like “To what extent do you believe [a neonatal nurse] interacts with [a neonatologist] in the electronic health record system to manage a patient?” This is asked for each collaborative relationship, and respondents are blind to the EHR-learned likelihood. The professionals were asked to choose one of the following five answers: “Not at all likely,” “Slightly likely,” “Moderately likely,” “Very likely,” and “Completely likely.” For statistical analysis, these survey responses were encoded as integer values (Likert score) in the range 1 to 5 (eg, “Not at all likely” is mapped to 1). The Likert scores were used to quantify an expert’s psychological expectations of latent collaborative relationships.

These surveys were distributed through the REDCap management system [37] and expert responses were requested after review and approval from the Vanderbilt Institutional Review Board (approval number: 191892). Using the survey results, we tested the following hypothesis: experts can distinguish latent collaborative relationships between high and low likelihood categories. We applied a linear regression model, shown in the following equation, to determine the Likert score for high and low likelihood relationships.

Likert Score=α+θ×β **(2)**

where θ {1 (high likelihood), 0 (low likelihood)} represents the high and low likelihoods of collaborative relationships identified from EHRs. Under this model, the Likert score for a low likelihood collaboration is α (θ=0) and for a high likelihood collaboration is α + β (θ=1). As such, the value of β corresponds to the difference of Likert scores for high and low likelihood collaborative relationships.

We used the Likert scores as observations to infer β via linear regression models. We then used ANOVA to test the significance of β≠0 against a null hypothesis β=0. We tested the hypothesis at the two-sided α=.05 significance level.

### Mining Weekday and Weekend Temporal Trends of Concurrent EHR Usage

We analyzed when (eg, shifts) concurrent sessions occur in EHRs. We assumed HCWs have different EHR interaction patterns during weekdays and weekends, and that those patterns are also different in the phases of a patient’s stay in the NICU. Therefore, we modeled weekday- and weekend-temporal trends of concurrent EHR usage, which we called temporal patterns, and focused on the following three specific phases of a patient’s hospital stay: admission, discharge, and intermediate phases.

Since all investigated patients had admission and discharge dates, we learned temporal patterns 24 hours after admission and 24 hours before discharge based on all those patients. We created the following four patient groups: (1) patients admitted during weekdays, (2) patients admitted during weekends, (3) patients discharged during weekdays, and (4) patients discharged during weekends. For a single patient in a patient group, we measured the number of concurrent intervals performed by HCWs on EHRs of that patient in each hour during the 24-hour window. Next, we calculated the average number in each hour for all patients in a group to form a temporal pattern. We used Wilcoxon rank-sum test to measure differences in the temporal patterns between weekdays and weekends because the Wilcoxon rank-sum test is based solely on the order in which the observations from the two patterns fall.

We chose days surrounding the middle of a patient’s hospital stay to represent the intermediate phase for the measurement of temporal patterns. We also separated patient stay into the following two subgroups: weekday and weekend stay. We measured the average number of concurrent intervals for each hour and used the Wilcoxon rank-sum test to assess the differences between weekday and weekend temporal patterns.

We also compared the differences between weekdays and weekends in the degree of concurrent EHR usage during the admission, discharge, and intermediate phases of hospital stay using *t* tests. This was done to determine if there was a significant difference between the means of two patterns, without the consideration of pattern observation order.

### Clustering Concurrent Intervals to Describe a Concurrent Session

The concurrent interval-action matrix recorded the number of times an action appeared in a concurrent interval. This matrix was used to learn similarities between concurrent intervals and concurrent sessions in terms of their affiliated action types. We performed principal component analysis (PCA), t-distributed stochastic neighbor embedding (t-SNE), and K-means clustering to aggregate intervals described by a cluster-concurrent interval matrix. The cluster-concurrent interval matrix was used jointly with the concurrent interval-concurrent session matrix to determine if a concurrent session contains intervals assigned to the same cluster or different clusters. This joint analysis was performed by calculating the dot product of the matrices. Such an analysis can highlight the complexity (eg, a concurrent session affiliated with a single cluster or multiple clusters) of a concurrent session.

### Availability of Data

The data sets that were generated and analyzed in this study are not publicly available because they include patients’ private information. However, the data sets can be obtained from the corresponding author upon reasonable request.

## Results

### Case Studies in the NICU

We gathered 4 months of EHR audit log data from a large academic medical center’s NICU. The data set contained 2,840,249 actions performed by 3303 HCWs (approximately 22,319 HCWs in the VUMC EHR system) to EHRs of 382 NICU patients. In this case study, we identified 2 minutes as the cutoff threshold in creating intervals from series of events; this was the point of maximum curvature, or “knee point,” determined through the Kneedle algorithm (as shown in Multimedia Appendix 1). We used 15 minutes as the threshold to separate inactive and active workdays, as determined through NICU expert questionnaires regarding EHR utilization. From these thresholds, we created 624,192 concurrent intervals, each of which comprised of consecutive sequences of 650 unique actions. There were 173,436 concurrent sessions created from the concurrent intervals.

### Examining Differences in the Concurrent Intensity Between NICU HCWs

We compared the concurrent intensities across the top 13 specialties having the highest average EHR times on active workdays. The 13 specialties consisting of 552 HCWs are listed in Table 2, along with their mean values and 95% CIs for concurrent intensity and EHR time. The concurrent intensity was calculated after excluding activities on inactive workdays. The statistical test results showed that there were significant differences in the EHR time (from mean 23.38, 95% CI 21.97-24.80, to mean 54.78, 95% CI 40.43-69.13; *P*<.001) and concurrent intensity (from mean 0.07, 95% CI 0.06-0.08, to mean 0.36, 95% CI 0.18-0.54; *P*<.001 with respect to the proportion of concurrent intervals and from mean 0.32, 95% CI 0.20-0.44, to mean 0.76, 95% CI 0.51-1.00; *P*<.001 with respect to the proportion of EHR time spent on concurrent intervals) between the 13 investigated specialties. We found that there were no significant relationships between EHR time and the proportion of time spent on concurrent intervals, except for extracorporeal membrane oxygenation (ECMO) respiratory therapists (*P*<.001). ECMO respiratory therapists had positive associations between time spent in EHRs and the proportion of time spent on concurrent intervals. This indicates that ECMO respiratory therapists work (76% of their EHR time) in a highly concurrent environment.

**Table 2:** Data for health care workers affiliated with 13 specialties.

Specialty	EHR^a^ time (min), mean (95% CI)	Number of event actions per day, mean (95% CI)	Proportion of concurrent intervals, mean (95% CI)	Proportion of EHR time spent on concurrent intervals, mean (95% CI)
MRI^b^-technologists	54.78 (40.43-69.13)	66.49 (54.82-78.16)	0.25 (0.07-0.44)	0.51 (0.17-0.85)
Diagnostic radiology-technologists	50.06 (39.77-60.35)	72.53 (62.64-82.41)	0.36 (0.18-0.54)	0.60 (0.39-0.80)
Pediatric cardiac ICU^c^-registered nurse	49.35 (45.02-53.69)	144.03 (134.77-153.29)	0.13 (0.08-0.18)	0.59 (0.48-0.69)
NICU^d^-registered nurse	36.56 (35.17-37.95)	98.86 (96.95-100.77)	0.07 (0.06-0.08)	0.40 (0.37-0.44)
Pediatrics-resident physician	34.03 (30.71-37.36)	121.58 (114.72-128.45)	0.08 (0.05-0.11)	0.63 (0.55-0.71)
Float pool-registered nurse	33.47 (26.06-40.88)	86.33 (75.05-97.62)	0.09 (0.02-0.15)	0.32 (0.20-0.44)
Inpatient-nurse practitioner	31.36 (28.75-33.98)	131.74 (124.82-138.66)	0.14 (0.09-0.18)	0.66 (0.58-0.75)
ECMO^e^-registered nurse	30.73 (21.93-39.52)	91.88 (75.31-108.46)	0.21 (0.04-0.38)	0.70 (0.39-1.00)
ECMO-respiratory therapist	30.62 (22.58-38.65)	93.31 (77.85-108.77)	0.28 (0.06-0.50)	0.76 (0.51-1.00)
Perioperative services-registered nurse	27.89 (20.50-35.27)	90.43 (58.77-122.08)	0.16 (0.07-0.26)	0.68 (0.40-0.96)
Rx inpatient core-pharmacist	26.27 (20.22-32.33)	58.16 (53.18-63.14)	0.12 (0.07-0.18)	0.75 (0.52-0.98)
Anesthesiology-nurse anesthetist	24.91 (19.86-29.96)	105.08 (88.39-121.78)	0.15 (0.06-0.23)	0.63 (0.38-0.89)
Pediatrics-respiratory therapist	23.38 (21.97-24.80)	103.87 (98.58-109.16)	0.11 (0.07-0.15)	0.57 (0.46-0.69)

^a^EHR: electronic health record.

^b^MRI: magnetic resonance imaging.

^c^ICU: intensive care unit.

^d^NICU: neonatal intensive care unit.

^e^ECMO: extracorporeal membrane oxygenation.

### Examining Latent Collaboration Networks in the NICU

**Figure 3:**
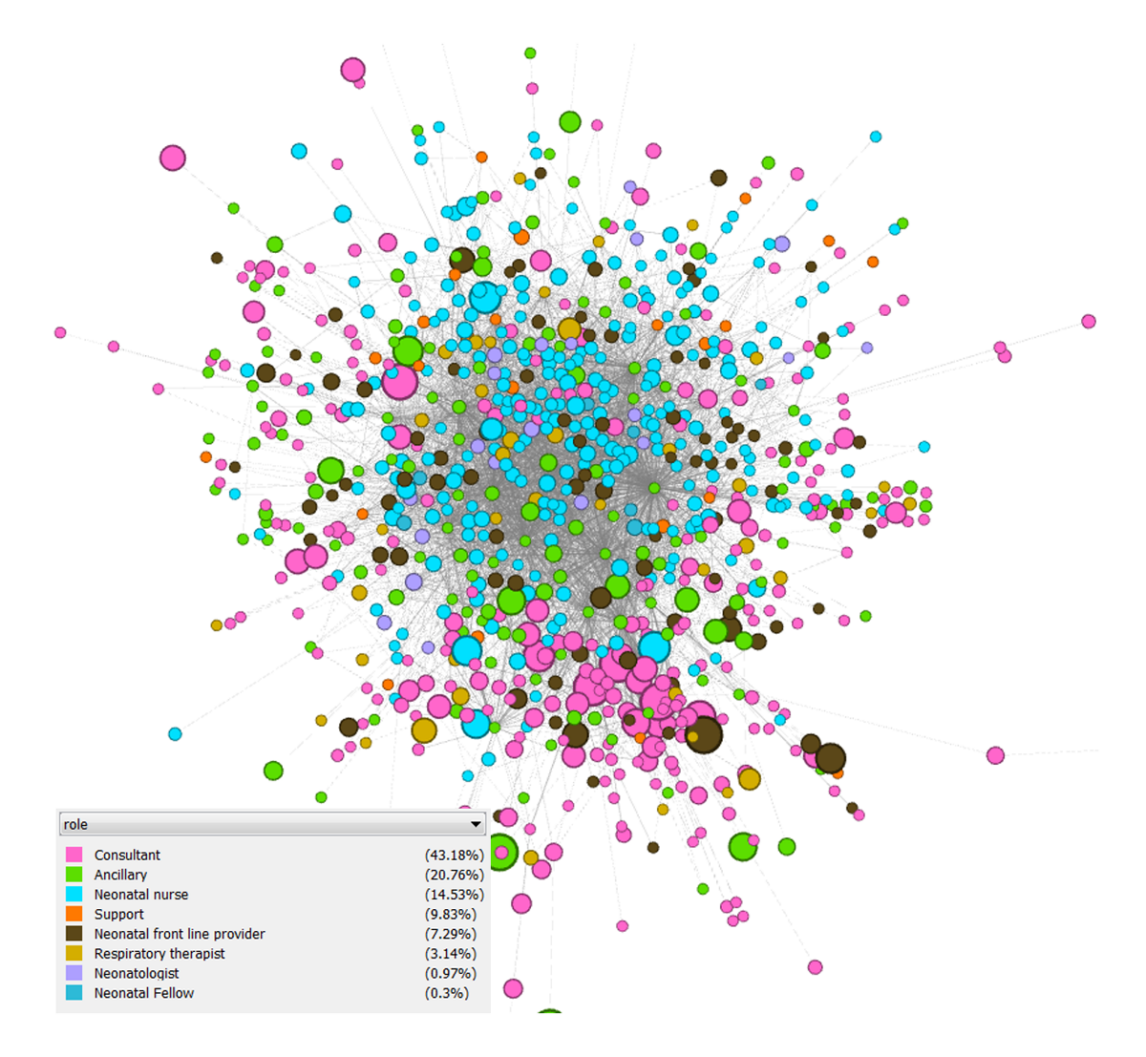
The latent collaboration network of HCWs. Each HCW is coded as a color based on their affiliated role category. The size of the node is determined by the proportion of time spent on the concurrent intervals over all intervals. A larger node size is associated with a higher proportion of time for concurrent EHR usage. EHR: electronic health record; HCW: health care worker.

**Figure 4:**
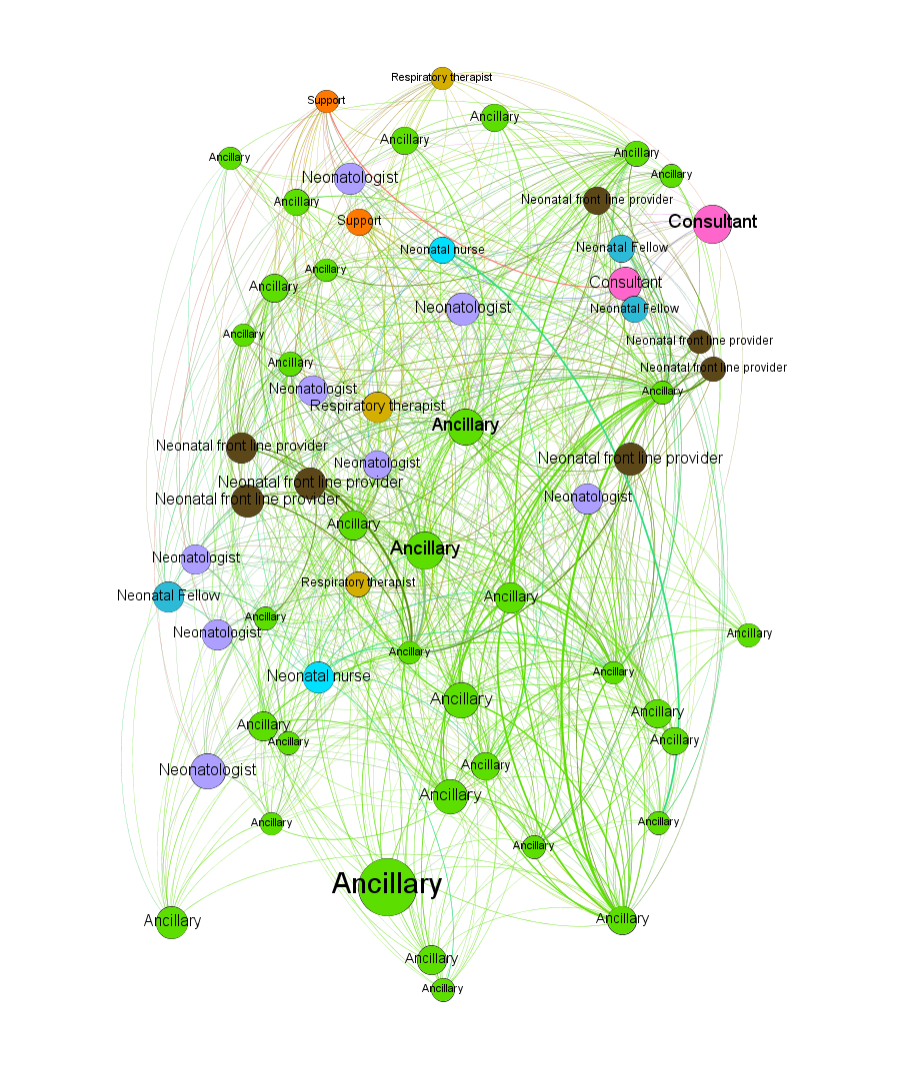
The 15-core HCW subnetwork. Each node is an HCW labeled by the roles. The size of the node is determined by the proportion of time spent on the concurrent intervals over all intervals. A larger node size is associated with a higher proportion of time for concurrent EHR usage. EHR: electronic health record; HCW: health care worker.

**Figure 5:**
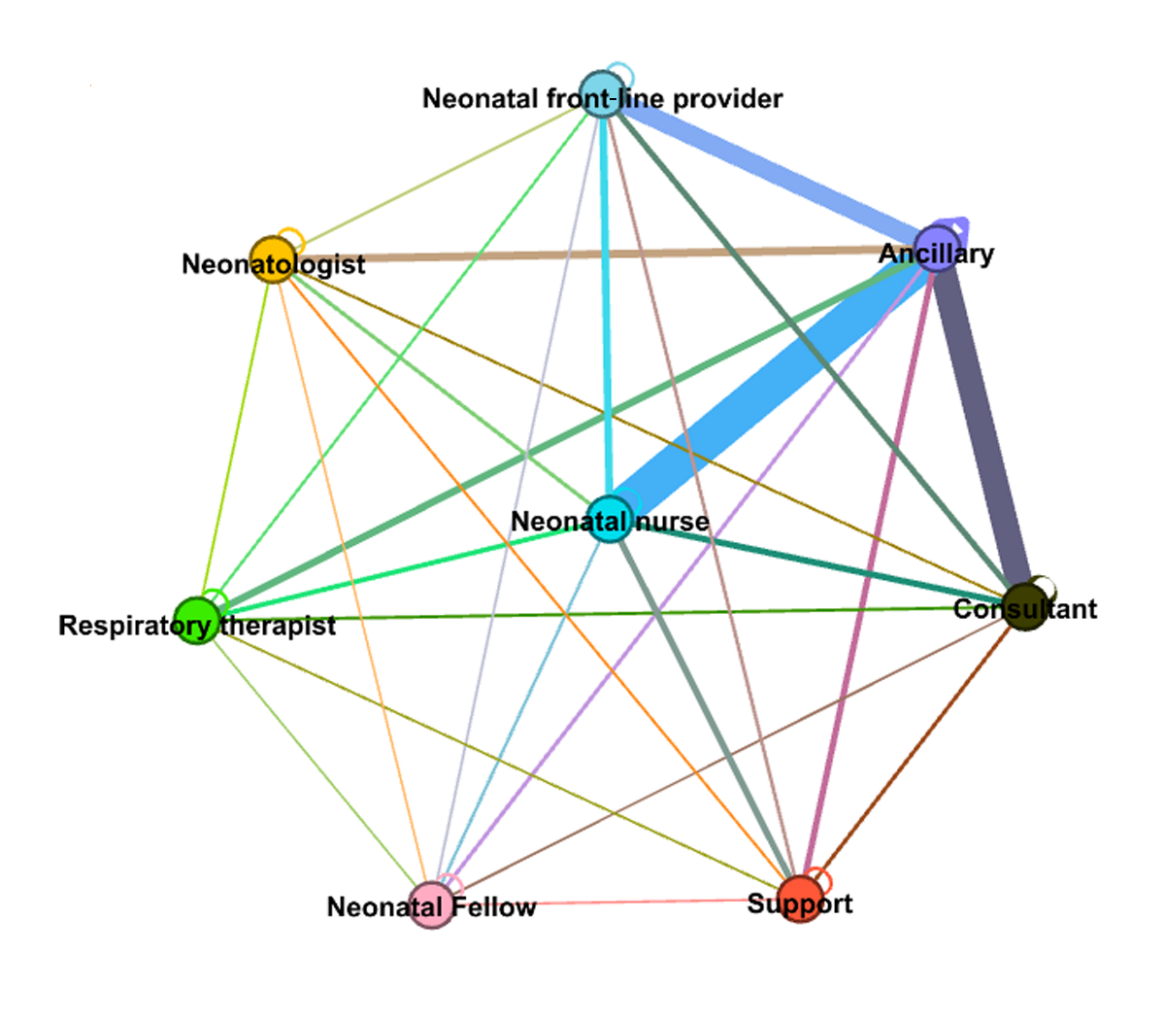
The collaboration network of the eight role categories. The nodes are roles. The weight of the edge indicates the strength of the collaboration.

We identified a collaboration network consisting of 857 HCWs with 4242 edges connecting them. The 857 HCWs were affiliated with 406 unique specialties. Figures 3 and 4 show the collaboration network of HCWs and its 15-core subnetwork, where each node is an HCW. The 15-core subnetwork was made up of 61 core HCWs, with 748 edges connecting those HCWs. Within the 15-core subnetwork, each HCW collaborated with at least 15 other HCWs. Compared with the full collaborative network (centered by a neonatal nurse), the 15-core subnetwork was centered by ancillary staff (latent collaboration with NICU professionals: neonatal nurses, neonatal frontline providers, neonatal fellows, neonatologists, and respiratory therapists). To interpret these collaborations among NICU HCWs, NICU experts categorized 406 specialties into the following eight roles: neonatologists, neonatal fellows, neonatal frontline providers (eg, nurse practitioners, physician assistants, hospitalists, and resident physicians), neonatal nurses, respiratory therapists, consultants (eg, surgeons, OB/GYN physicians, hematology physicians, radiology physicians, anesthesiologists, and genetics counselors), ancillary staff (eg, registered dietitians, social workers, case managers, technicians, and phlebotomists), and support staff (eg, clerks, information technology staff, coordinators, and medical assistants). The collaboration network, as shown in Figure 5, was visualized at the level of roles. The mappings between the eight roles and 406 specialties are presented in Multimedia Appendix 2.

Overall, ancillary staff and consultants had higher concurrent intensity (larger node size) than HCWs with other roles (Figure 3). Supporting this was the observation that ancillary staff nodes were distributed across the 15-core subnetwork (Figure 4). Figure 5 indicates the latent collaborative relationships among the eight professional roles. The relationships between ancillary HCWs, consultants, neonatal nurses, and neonatal frontline providers were strong. Neonatal nurses were very active in the network, often collaborating with HCWs from ancillary staff, which was the most collaborative role.

### Collaboration Validation Results

We sampled 12 (12/28) collaborative relationships (six of high and six of low likelihood), and generated a survey containing 12 questions (Multimedia Appendix 3). The total number of NICU experts who accepted the invitation and participated in the survey was 13, with four neonatologists, three neonatal fellows, three neonatal nurses, two nurse practitioners, and one respiratory therapist. The 13 experts, including neonatal attendings, nurses, nurse practitioners, residents, fellows, and respiratory therapists, were representatives of the expertise in the NICU. The average number of years those experts have been working at the NICU is 5.65. All 13 responding NICU experts completed the survey (100% response rate). The number of years of experts working in the NICU is depicted in Multimedia Appendix 4. The results of the Likert scores are shown in Multimedia Appendix 5. Our assumption of using a linear regression model was confirmed by the quantile-quantile plot, as shown in Figure S2 in Multimedia Appendix 5. Overall, NICU experts could distinguish collaborative relationships between high and low likelihoods (β=.88, Likert score: 3.54, 95% CI 3.31-4.37 vs 2.64, 95% CI 2.46-3.29; *P*<.001). The Likert scores of the 12 collaborations surveyed from NICU experts are shown in Table 3.

**Table 3:** Likert scores of the 12 investigated collaborative relationships.

Index and collaborative relationship			Likert score from NICU^a^ professionals
**Collaborative relationships with high likelihoods learned from EHRs^b^**			
	1: Neonatal front line provider ↔ Consultant	3.85	
	2: Ancillary staff ↔ Consultant	2.23	
	3: Neonatal nurse ↔ Consultant	4.46	
	4: Neonatal front line provider ↔ Ancillary staff	4.08	
	5: Ancillary staff ↔ Neonatal nurse	3.46	
	6: Support staff ↔ Neonatal nurse	3.15	
**Collaborative relationships with low likelihoods learned from EHRs**			
	7: Neonatal front line provider ↔ Neonatologist	3.15	
	8: Ancillary staff ↔ Support staff	2.07	
	9: Neonatal nurse ↔ Neonatal fellow	3.07	
	10: Neonatal front line provider ↔ Neonatal fellow	2.84	
	11: Ancillary staff ↔ Neonatal fellow	3.07	
	12: Support staff ↔ Neonatal fellow	1.69	

^a^NICU: neonatal intensive care unit.

^b^EHR: electronic health record.

### Temporal Trends of Concurrent EHR Usage

**Figure 6:**
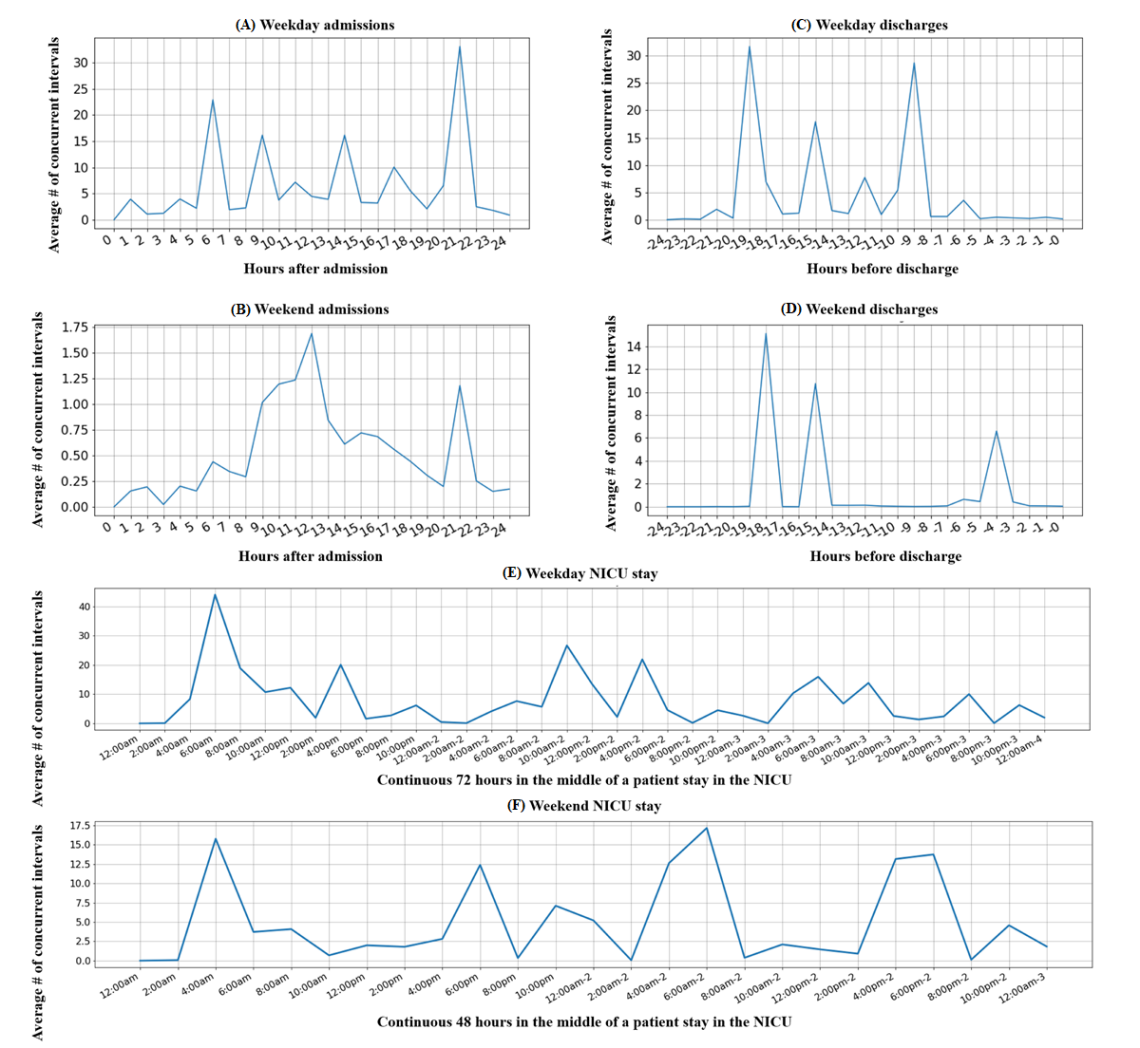
Concurrent EHR usage temporal trends 24 hours after admission (A and B), 24 hours before discharge (C and D), and consecutive intermediate days of hospital stay. The trends are measured from weekdays and weekends. EHR: electronic health record; NICU: neonatal intensive care unit.

Figure 6 shows the temporal patterns 24 hours after admission (6A and 6B) and 24 hours before discharge (6C and 6D). These patterns were separated by weekday or weekend status. The temporal patterns were significantly different for both admission (average number of concurrent intervals per hour: 11.60 vs 0.54, *P*<.001) and discharge days (4.72 vs 1.45, *P*<.001), but not for the intermediate phase of hospital stay.

Expectedly, there was more concurrent EHR usage between HCWs on weekdays than weekends across all three phases of hospital stay (average number of concurrent intervals per hour: 9.56 vs 2.34, *P*<.001).

### Clusters of Concurrent Intervals and the Composition of a Concurrent Session

**Figure 7:**
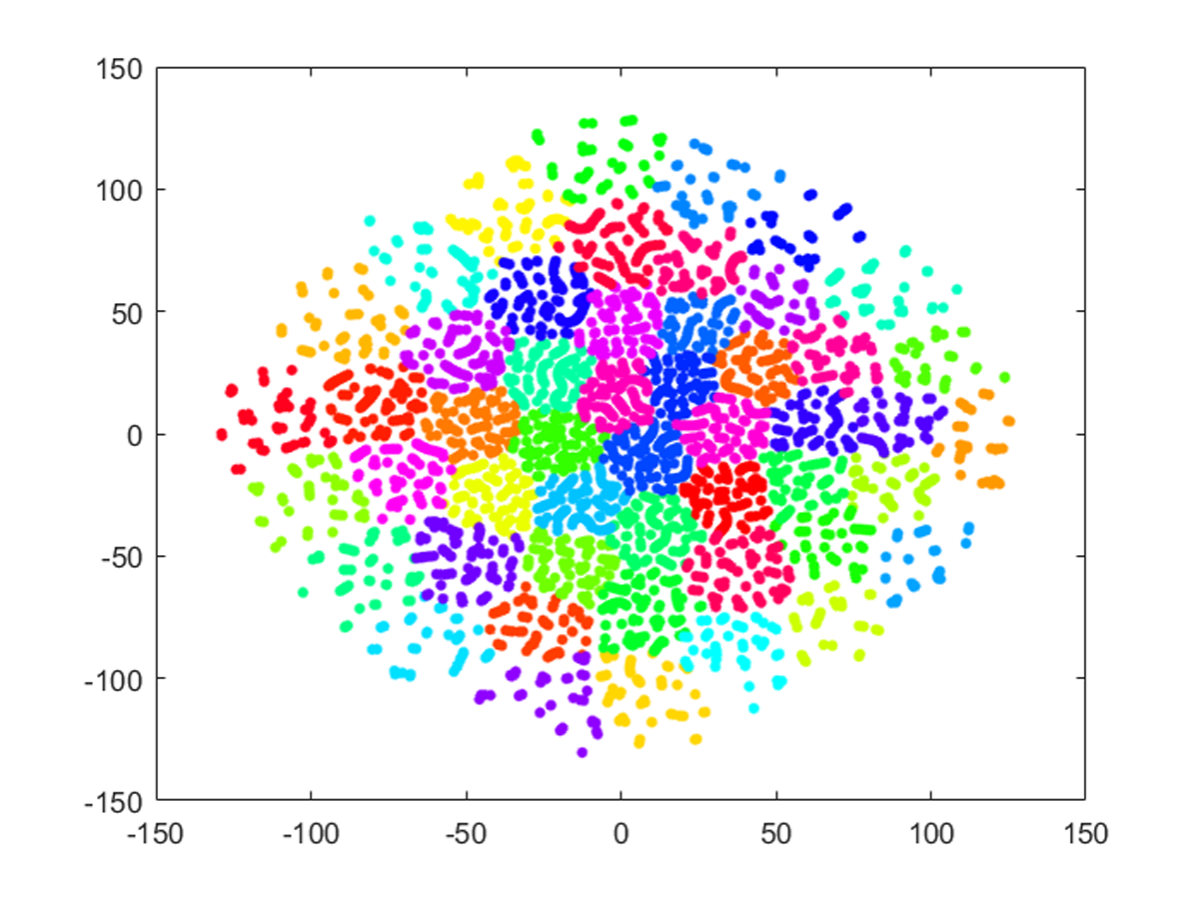
A visualization of the 50 clusters of concurrent intervals. Each node is a concurrent interval, and each color indicates the cluster group to which an interval belongs. The axes are t-distributed stochastic neighbor embedding–reduced components.

We clustered the concurrent intervals using their constituent actions as features. We used PCA to reduce the dimensionality to the top 10 components, which explained 97% of the variance. We then applied t-SNE on the 10 PCA components to further reduce the data to two dimensions. Finally, we used the k-means clustering algorithm to form 50 clusters, as shown in Figure 7. This K of 50 was determined by minimizing the total within-cluster sum of squared errors (WSS). The squared error for each point was the square of the distance of the point from its predicted cluster center. The WSS score was the sum of these squared errors for all the points. The plot of WSS versus k is depicted in Figure S1 in Multimedia Appendix 6. As shown in Figure 7, the 50 clusters were well separated. Concurrent intervals within each cluster shared similar actions.

Using the clusters visualized in Figure 7, we examined intercluster relationships, as shown in Figure S2 in Multimedia Appendix 6. The calculated intercluster network described the pairwise relationship of each of the concurrent sessions.

**Figure 8:**
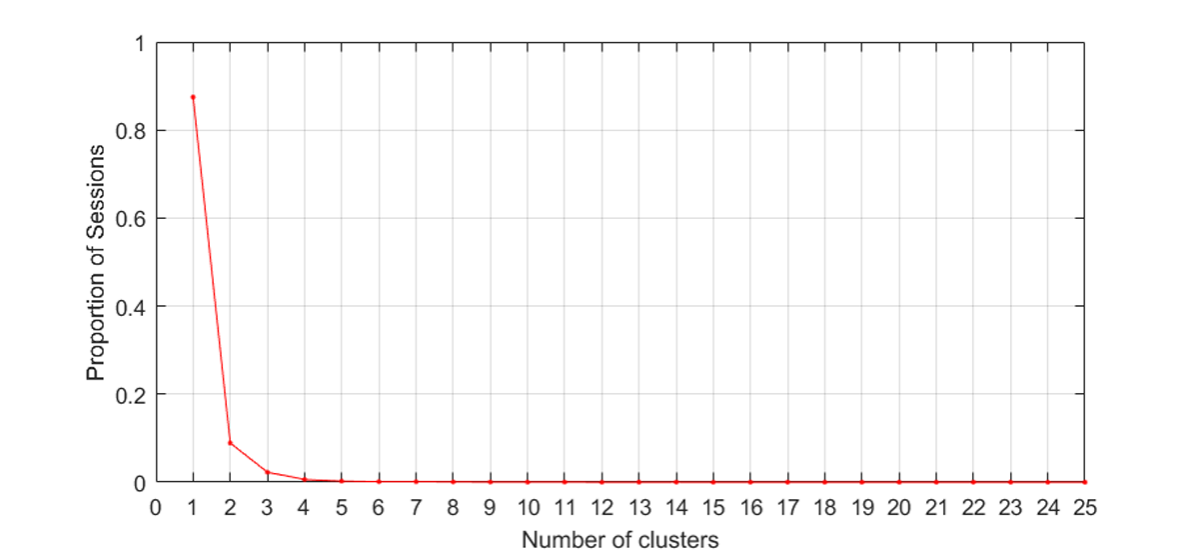
The distribution of concurrent sessions as a function of the number of clusters that concurrent intervals are affiliated with.

Figure 8 shows the distribution of concurrent sessions in terms of the number of clusters affiliated. We showed that over 87% of concurrent sessions could be unambiguously assigned into a single unique cluster, indicating that most HCWs perform similar actions in a concurrent session. About 13% of concurrent sessions, consisting of concurrent intervals, came from multiple clusters.

Our unsupervised learning framework could identify and quantify concurrent EHR usage from audit log data. Based on concurrent EHR usage, we could determine the proportion of concurrent activities, the proportion of time spent on those activities, HCWs who participate in concurrent or latent interactions, the temporal trends of concurrent EHR usage on weekdays and weekends in the three phases of hospital stay, and the complexity of concurrent activities (single cluster vs multiple clusters).

## Discussion

### Principal Findings

We presented a novel framework to measure latent collaboration from EHR audit logs, and we established novel metrics, which may be useful for the analysis of latent HCW collaboration. EHR system usage is pervasive and still increasing. While there are studies that measured collaboration, few targeted the growing paradigm of latent collaboration among HCWs. We demonstrated the use of our informatics framework in the analysis of latent collaboration. We examined the concurrent intensity across various HCW specialties and found that there was a statistically significant difference in the proportion of concurrent activities and the proportion of time spent on those activities. It was noted that in some settings, clinicians shared the same workstation or computer terminal. Concurrent EHR usage may have highly variable ergonomics between health care settings, for example, in some instances, HCWs may have to share one workstation, making concurrent EHR usage impossible. In this study, we identified latent collaboration among HCWs coming from various departments, and thus, there was a low probability for HCWs sharing the same workstation or computer terminal. If latent collaboration is identified among HCWs from the same department or unit, it would be better for HCOs to allocate more workstations or computer terminals to HCWs within the department/unit to achieve high performing collaboration in EHR systems.

We examined networks that represented the collaborative relationships between HCWs (Figure 2). By using our framework, we identified HCW relationships between defined role categories in the NICU. We assessed our framework in a NICU setting, and it demonstrated the effectiveness of using concurrent EHR usage measuring latent collaboration. Based on the observations from Figures 3 to 5, EHR vendors or HCOs may need to establish communication channels in EHR systems for ancillary staff to collaborate with other HCWs (eg, NICU nurses) to deliver high quality care for neonates.

Strikingly, strong collaborative relationships between consultants, ancillary staff, and neonatal nurses are described by our framework (Figure 4), though NICU experts do not consistently assign collaborative relationships between them (eg, collaborative relationship between ancillary staff and consultants) (Table 3). One potential reason for this discrepancy is that our survey respondents were not part of ancillary staff or consultant roles, thus limiting the description of these specific collaborations. Recruiting HCWs from these roles as survey respondents remains high priority, but is challenging due to their assignments to heterogeneous departments and care units. We believe a large scale study is required to formally assess latent collaborative relationships between ancillary staff and consultants.

We examined concurrent EHR usage patterns in the admission, discharge, and intermediate phases of hospital stay, finding significant differences in patterns between weekdays and weekends. This suggests that HCWs act differently on weekdays and weekends, which may assist HCOs in using different staffing strategies optimizing latent collaboration on weekdays and weekends.

We clustered the concurrent intervals of HCWs and highlighted their interconnectivity (Multimedia Appendix 6). These clusters and their neighbors may be used to reduce the search space for the analysis of audit log data. Potentially, this enables higher throughput process mining or the targeting of specific dominant HCW roles.

### Scope of This Study and Its Limitations

This was a pilot study, and we would like to acknowledge some limitations that may guide prospective latent collaboration-related studies. Using concurrent HCW activity can help HCOs or EHR vendors identify potential collaborative relationships among HCWs; however, such relationships need to be further validated when optimizing or refining EHR systems. Moreover, causative explanations for these latent relationships are not determined. We believe that describing the causes for certain collaborations would require additional data and further investigations on the HCW-EHR system interaction workflow. This study does not describe the cause of this phenomenon, but highlights its existence and provides an avenue of hypothesis generation for future work.

There are multiple forms of collaboration between HCWs [26]. Collaboration may consist of direct and explicit physical communication or latent interactions through digital platforms, but our study focused on latent interactions involving EHR systems. Learning broader forms of collaboration requires the integration of a broader range of data resources.

We investigated when concurrent EHR usage occurs, but did not investigate the underlying causes for the observed differences. Our focus on concurrent EHR usage may not be able to detect collaborative activities that do not have time overlaps. Further, we acknowledge that not every piece of concurrent HCW activity indicates a latent collaboration. It is possible that overlapping usage of the same target patient EHR is coincidental. For instance, some HCWs may simply have overlapping shifts, which may be detected as false positives with our framework, thus requiring further validation to flag these scenarios.

Since interval durations were calculated through the difference of timestamps, we did not capture the duration of interval-ending actions. Potential remedies in logging the durations of these types of actions include the use of video monitoring to track HCW activities in EHRs.

NICU experts distinguished latent collaborative relationships between high and low likelihoods learned from EHRs; however, we did not assess the plausibility of each inferred latent collaborative relationship at the level of the EHR user (edges in Figure 3).

Finally, we used a threshold determined by experts to define active EHR workdays. Activities occurring on inactive EHR workdays may also contribute evidence for measuring latent collaborative relationships. Moreover, categorization of 406 specialists named by the Epic system into eight general roles was conducted by NICU experts, which may be biased according to their expertise and experiences.

### Conclusion

We presented an informatics framework relying on concurrent EHR usage to learn latent collaboration. We explored the advantages of the framework by conducting the following four types of analyses: (1) quantifying time spent interacting with EHRs and on concurrent usage, (2) investigating the latent collaborative relationships among HCWs engaging in highly concurrent EHR usage, (3) measuring temporal trends of concurrent EHR usage on weekdays and weekends in the three phases of hospital stay, and (4) clustering EHR activities to describe the complexity of concurrent EHR usage. We assessed the effectiveness of our framework through a case study and anticipated that its generalizability will further enable the analysis of how latent collaborative interactions affect patient care, discharge times, and clinician workload, stress, or burnout.

## Appendix

Multimedia Appendix 1 Determination of the interval time threshold.

Multimedia Appendix 2 Categorization of specialists into roles.

Multimedia Appendix 3 Survey questions.

Multimedia Appendix 4 Summary statistics of neonatal intensive care unit experts participating in online surveys.

Multimedia Appendix 5 Likert scores of 12 collaboration relationships surveyed from neonatal intensive care unit experts, and the plot of standardized residuals for the linear regression model.

Multimedia Appendix 6 Determination of the number of clusters, and visualization of the cluster-cluster relationships.

## Abbreviations

ANOVA: analysis of variance
ECMO: extracorporeal membrane oxygenation
EHR: electronic health record
HCO: health care organization
HCW: health care worker
NICU: neonatal intensive care unit
PCA: principal component analysis
t-SNE: t-distributed stochastic neighbor embedding
WSS: within-cluster sum of squared errors
