# Supplementary material for: Measuring Collaboration Through Concurrent Electronic Health Record Usage: Network Analysis Study
Source: JMIR Med Inform. 2021 Sep 3;9(9):e28998. doi: 10.2196/28998 (PMC8449299; doi:10.2196/28998)
Supplement: Multimedia Appendix 1 [file medinform_v9i9e28998_app1.docx]

**Multimedia Appendix 1.** Determination of the interval time threshold.


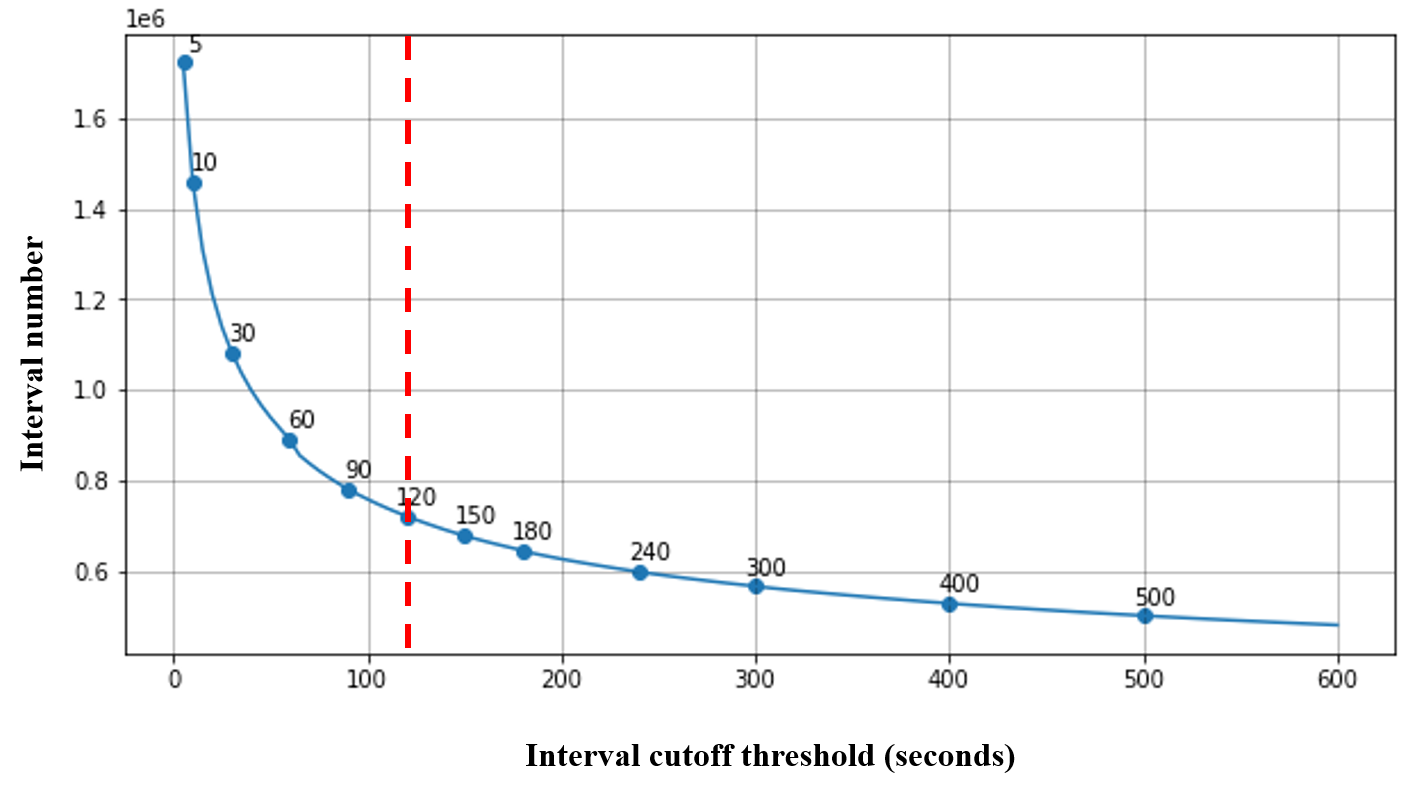


The data-driven detection of a stable interval creation threshold by finding the point of maximum curvature in the interval number curve. This curve was derived through testing 12 thresholds, which yielded a diminishing number of intervals, each represented by a labeled point ranging from 5 to 600 seconds. Here, the selected interval time threshold is highlighted in red, being 120 seconds, and was the point of maximum curvature estimated through the Kneedle algorithm.
