# Supplementary material for: Measuring Collaboration Through Concurrent Electronic Health Record Usage: Network Analysis Study
Source: JMIR Med Inform. 2021 Sep 3;9(9):e28998. doi: 10.2196/28998 (PMC8449299; doi:10.2196/28998)
Supplement: Multimedia Appendix 2 [file medinform_v9i9e28998_app2.docx]

**Multimedia Appendix 2.** Categorization of specialists into roles.

To help audiences understand the collaborations among NICU HWs, we categorize 406 specialties into the following 8 roles.

1. Neonatologist
2. Neonatal fellow
3. Neonatal front line provider (e.g., nurse practitioner, physician assistant, hospitalist, or resident physician)
4. Neonatal nurse
5. Respiratory therapist
6. Consultant
7. Ancillary staff (e.g., registered dietitian, social worker, case manager, technician, phlebotomist)
8. Support staff (e.g., clerk, IT staff, financial and administrative, supply chain)

**Table S1.** The 406 specialties are categorized into 8 roles.

| **Role** | **Specialty** |
| --- | --- |
| Ancillary | Allied Health Non-Employee-ANCILLARY STUDENT |
| Ancillary | Audiology-ANCILLARY STUDENT |
| Ancillary | Audiology-AUDIOLOGIST |
| Ancillary | Blood Bank-ANCILLARY STUDENT |
| Ancillary | Blood Bank-PHLEBOTOMIST |
| Ancillary | Child Life Services-CHILD LIFE SPECIALIST |
| Ancillary | Children's Hospital Administration-PHARMACIST |
| Ancillary | Children's Hospital Administration-SOCIAL WORKER |
| Ancillary | Children's Hospital Nutrition Clinic-REGISTERED DIETITIAN |
| Ancillary | Children's Hospital Nutrition Support Srvcs-ANCILLARY STUDENT |
| Ancillary | Children's Hospital Nutrition Support Srvcs-REGISTERED DIETITIAN |
| Ancillary | Children's Hospital Oto Audio Speech-LICENSED NURSE |
| Ancillary | Children's Hospital Oto Audio Speech-MEDICAL ASSISTANT |
| Ancillary | Children's Hospital Rx Clinical Services-PHARMACIST |
| Ancillary | Children's Hospital Rx Inpatient Core-PHARMACIST |
| Ancillary | Children's Hospital Rx Leadership-PHARMACIST |
| Ancillary | Children's Hospital Rx OR Services-PHARMACIST |
| Ancillary | Children's Hospital Rx Outpatient Services-PHARMACIST |
| Ancillary | Children's Hospital Rx Outpatient Services-PHARMACY INTERN |
| Ancillary | Children's Hospital Rx Satellite Services-PHARMACIST |
| Ancillary | Children's Hospital Rx Services & Education-PHARMACY INTERN |
| Ancillary | Clinical Research Center-REGISTERED NURSE |
| Ancillary | Contact Center-PHLEBOTOMIST |
| Ancillary | Core - Hematology-DIETICIAN STUDENT |
| Ancillary | Core - Hematology-PHLEBOTOMIST |
| Ancillary | Cytogenetics-PHLEBOTOMIST |
| Ancillary | General Pediatrics-RESEARCH COORDINATOR - NON CLINICAL |
| Ancillary | Hearing And Speech-ANCILLARY STUDENT |
| Ancillary | Hearing And Speech-AUDIOLOGIST |
| Ancillary | Hearing And Speech-SPEECH AND LANGUAGE PATHOLOGIST |
| Ancillary | Hearing School-SOCIAL WORKER |
| Ancillary | Hematopathology-PHLEBOTOMIST |
| Ancillary | Home Care Services Admin-MEDICAL ASSISTANT |
| Ancillary | Medical Speech - Peds-SPEECH AND LANGUAGE PATHOLOGIST |
| Ancillary | Microbiology-PHLEBOTOMIST |
| Ancillary | Molecular Infectious Disease-PHLEBOTOMIST |
| Ancillary | Murfreesboro Recovery-REGISTERED NURSE |
| Ancillary | Pastoral Care-SPIRITUAL CARE |
| Ancillary | Pathology |
| Ancillary | Pathology |
| Ancillary | Pathology-PHLEBOTOMIST |
| Ancillary | Patient & Family-ART THERAPIST |
| Ancillary | Patient & Family-MUSIC THERAPIST |
| Ancillary | Patient & Family-SPIRITUAL CARE |
| Ancillary | Pediatric Emergency -PARAMEDIC |
| Ancillary | Pediatric Hematology-RESEARCH COORDINATOR - NON CLINICAL |
| Ancillary | Pediatric Social Work-SOCIAL WORKER |
| Ancillary | Peds Rehab-OCCUPATIONAL THERAPIST |
| Ancillary | Peds Rehab-PHYSICAL THERAPIST |
| Ancillary | Pharm Cln Investigational Drug-PHARMACIST |
| Ancillary | Pharm Inpt Sterile Products-PHARMACIST |
| Ancillary | Pharmacy Clinical Operations-PHARMACIST |
| Ancillary | Pharmacy Clinical Programs-PHARMACIST |
| Ancillary | Pharmacy Inpt Central Svcs-PHARMACIST |
| Ancillary | Pharmacy Inpt Operations-PHARMACIST |
| Ancillary | Physical Therapy Out-PHYSICAL THERAPIST |
| Ancillary | Quality and POCT Admin-PHLEBOTOMIST |
| Ancillary | Quality and Safety-PHLEBOTOMIST |
| Ancillary | Special Chemistry-PHLEBOTOMIST |
| Ancillary | Specimen Processing-PHLEBOTOMIST |
| Ancillary | Specimen Receiving-PHLEBOTOMIST |
| Ancillary | Student Health Clinical-PHLEBOTOMIST |
| Ancillary | Transfusion Admin-PHLEBOTOMIST |
| Ancillary | Children's Hospital Cath Lab-REGISTERED NURSE |
| Ancillary | Core Lab - 2nd Shift-PHLEBOTOMIST |
| Ancillary | Core Lab - 3rd Shift-PHLEBOTOMIST |
| Ancillary | Laboratory - Clinical-PHLEBOTOMIST |
| Ancillary | Reference Lab-PHLEBOTOMIST |
| Ancillary | Specialty Labs Admin-PHLEBOTOMIST |
| Ancillary | Virology Laboratory-PHLEBOTOMIST |
| Ancillary | Audiology-TECHNICIAN |
| Ancillary | Cancer Pharmacy-PHARMACY TECHNICIAN |
| Ancillary | Center for Women's Health-TECHNICIAN |
| Ancillary | Center for Women's Health-TECHNOLOGIST |
| Ancillary | Children's Hospital Clinics-TECHNICIAN |
| Ancillary | Children's Hospital Float Pool-TECHNICIAN |
| Ancillary | Children's Hospital Neuro & Neuro Surgery Competencies-TECHNICIAN |
| Ancillary | Children's Hospital Patient Safety-TECHNICIAN |
| Ancillary | Children's Hospital Rx Inpatient Tech-OCCUPATIONAL THERAPY ASSISTANT STUDENT |
| Ancillary | Children's Hospital Rx Inpatient Tech-PHARMACY TECHNICIAN |
| Ancillary | Children's Hospital Rx OR Services-PHARMACY TECHNICIAN |
| Ancillary | Children's Hospital Rx Outpatient Tech-PHARMACY TECHNICIAN |
| Ancillary | Children's Hospital Rx Satellite Services-PHARMACY TECHNICIAN |
| Ancillary | Children's Hospital Rx Services & Education-PHARMACY TECHNICIAN |
| Ancillary | Children's Hospital Sterile Processing-TECHNOLOGIST |
| Ancillary | Computed Tomography-TECHNOLOGIST |
| Ancillary | Data Manager-PHARMACY TECHNICIAN |
| Ancillary | Diagnostic Radiology-TECHNOLOGIST |
| Ancillary | GI Medicine-TECHNICIAN |
| Ancillary | HealthIT Pharmacy-PHARMACY TECHNICIAN |
| Ancillary | Hospital Float Pool-TECHNICIAN |
| Ancillary | Hospital Nutrition Clinic-TECHNICIAN |
| Ancillary | MRI-TECHNOLOGIST |
| Ancillary | Medical Center IT CAST-TECHNICIAN |
| Ancillary | Murfreesboro Admin-TECHNOLOGIST |
| Ancillary | Murfreesboro Diag Imag-TECHNOLOGIST |
| Ancillary | NICU-TECHNICIAN |
| Ancillary | Neurodiagnostic - EEG-TECHNICIAN |
| Ancillary | Nuclear Medicine-TECHNOLOGIST |
| Ancillary | Obstetrics-TECHNICIAN |
| Ancillary | Orthopaedic Institute Radiology-TECHNOLOGIST |
| Ancillary | Ped Heart Institute-TECHNICIAN |
| Ancillary | Ped Heart Institute-TECHNOLOGIST |
| Ancillary | Ped Urology-TECHNICIAN |
| Ancillary | Pediatric Emergency -TECHNICIAN |
| Ancillary | Pediatric Hematology/Oncology-TECHNICIAN |
| Ancillary | Pediatric Medicine Acute Care -TECHNICIAN |
| Ancillary | Pediatric Neurodiagnostics-TECHNICIAN |
| Ancillary | Pediatric Neurodiagnostics-TECHNOLOGIST |
| Ancillary | Pediatric Radiology Admin-TECHNOLOGIST |
| Ancillary | Pediatric Surgery-TECHNICIAN |
| Ancillary | Pharm Cln Investigational Drug-PHARMACY TECHNICIAN |
| Ancillary | Pharm Compliance & Proc Improv-PHARMACY TECHNICIAN |
| Ancillary | Pharmacy PAP Team-PHARMACY TECHNICIAN |
| Ancillary | Radiology-TECHNOLOGIST |
| Ancillary | Rehabilitation Pharmacy-PHARMACY TECHNICIAN |
| Ancillary | STAM-TECHNICIAN |
| Ancillary | Special Procedures-TECHNOLOGIST |
| Ancillary | Specialty Pharm-PHARMACY TECHNICIAN |
| Ancillary | Specimen Receiving-TECHNICIAN |
| Ancillary | Ultrasound-TECHNOLOGIST |
| Ancillary | Children's Hospital Radiology Access-TECHNICIAN |
| Ancillary | PCARD-TECHNICIAN |
| Ancillary | Pediatric Cardiac ICU-TECHNICIAN |
| Ancillary | Pediatric Cardiology-TECHNOLOGIST |
| Ancillary | Labor & Delivery-TECHNICIAN |
| Ancillary | Children's Hospital Rx Residents-PHARMACIST |
| Ancillary | PGY1 Residents Program-PHARMACIST |
| Ancillary | Children's Hospital Rx Residents-PHARMACY TECHNICIAN |
| Ancillary | Pathology-RESIDENT PHYSICIAN |
| Ancillary | Children's Hospital Case Management-CASE MANAGER |
| Ancillary | Children's Hospital Complex Care Competencies-CASE MANAGER |
| Ancillary | Children's Hospital Hem Onc Clinic-CASE MANAGER |
| Ancillary | Children's Hospital Hemoc Competencies-CASE MANAGER |
| Ancillary | Neonatal Services-CASE MANAGER |
| Ancillary | Ped Heart Institute-CASE MANAGER |
| Ancillary | Transition Management Office-CASE MANAGER |
| Consultant | Adult Ent Volunteer BBP-MEDICAL STUDENT |
| Consultant | Anesthesia Services-NURSE ANESTHETIST |
| Consultant | Anesthesiology-ANESTHESIOLOGIST |
| Consultant | Anesthesiology-NURSE ANESTHETIST |
| Consultant | CVICU-REGISTERED NURSE |
| Consultant | Cancer PCC -NURSE PRACTITIONER |
| Consultant | Cancer Pharmacy-PHARMACIST |
| Consultant | Center for Women's Health-REGISTERED NURSE |
| Consultant | Children's Hospital GI-REGISTERED NURSE |
| Consultant | Children's Hospital Hem Onc Clinic-REGISTERED NURSE |
| Consultant | Children's Hospital Hemoc Competencies-REGISTERED NURSE |
| Consultant | Children's Hospital Holding Room/ PACU-REGISTERED NURSE |
| Consultant | Children's Hospital Neuro & Neuro Surgery Competencies-LICENSED NURSE |
| Consultant | Children's Hospital Neuro & Neuro Surgery Competencies-REGISTERED NURSE |
| Consultant | Children's Hospital Nursing-WOUND/OSTOMY CERTIFIED NURSE |
| Consultant | Children's Hospital Perioperative Srvcs-LICENSED NURSE |
| Consultant | Children's Hospital Perioperative Srvcs-REGISTERED NURSE |
| Consultant | Developmental Medicine-NURSE PRACTITIONER |
| Consultant | ECMO-REGISTERED NURSE |
| Consultant | ECMO-RESPIRATORY THERAPIST |
| Consultant | Emergency Services-REGISTERED NURSE |
| Consultant | Heart Transplant-REGISTERED NURSE |
| Consultant | Hematology Oncology-REGISTERED NURSE |
| Consultant | Internal Medicine-REGISTERED NURSE |
| Consultant | MICU-REGISTERED NURSE |
| Consultant | Medicine O/P-REGISTERED NURSE |
| Consultant | Neuro Surgery-NURSE PRACTITIONER |
| Consultant | Ob-Gyn-GENETIC COUNSELOR |
| Consultant | Ob-Gyn-MIDWIFE |
| Consultant | Ob-Gyn-NURSE PRACTITIONER |
| Consultant | Obstetrics-REGISTERED NURSE |
| Consultant | Office of Advanced Practice-NURSE ANESTHETIST |
| Consultant | Ophthalmology-REGISTERED NURSE |
| Consultant | Otolaryngology-MEDICAL ASSISTANT |
| Consultant | Otolaryngology-NURSE PRACTITIONER |
| Consultant | PICU-REGISTERED NURSE |
| Consultant | Ped Heart Institute-REGISTERED NURSE |
| Consultant | Ped Ortho-ATHLETIC TRAINER |
| Consultant | Ped Ortho-REGISTERED NURSE |
| Consultant | Ped Urology-REGISTERED NURSE |
| Consultant | Pediatric Critical Care-ANESTHESIOLOGIST |
| Consultant | Pediatric Emergency -REGISTERED NURSE |
| Consultant | Pediatric Genetics-GENETIC COUNSELOR |
| Consultant | Pediatric Genetics-NURSE PRACTITIONER |
| Consultant | Pediatric Hematology/Oncology-REGISTERED NURSE |
| Consultant | Pediatric Hospital Medicine-NURSE PRACTITIONER |
| Consultant | Pediatric Infectious Disease-RESEARCH COORDINATOR - NON CLINICAL |
| Consultant | Pediatric Medicine Acute Care -CNA |
| Consultant | Pediatric Medicine Acute Care -COORDINATOR |
| Consultant | Pediatric Medicine Acute Care -REGISTERED NURSE |
| Consultant | Pediatric Perfusion Group-PERFUSIONIST |
| Consultant | Pediatric Pulmonary-NURSE PRACTITIONER |
| Consultant | Pediatrics-MEDICAL ASSISTANT |
| Consultant | Perioperative Admin-REGISTERED NURSE |
| Consultant | Psychiatric Hospital Adol CSL-REGISTERED NURSE |
| Consultant | SICU-REGISTERED NURSE |
| Consultant | Urology-LICENSED NURSE |
| Consultant | Urology-NURSE PRACTITIONER |
| Consultant | Vascular Institute Nurse Practitioners-NURSE PRACTITIONER |
| Consultant | Visceral Adipose Tissue-REGISTERED NURSE |
| Consultant | Cardiology-REGISTERED NURSE |
| Consultant | Cardiovascular Medicine-RESEARCH COORDINATOR - NON CLINICAL |
| Consultant | Cardiovascular Medicine-RESOURCE |
| Consultant | Children's Hospital Cardiac OR-LICENSED NURSE |
| Consultant | Children's Hospital Cardiac OR-REGISTERED NURSE |
| Consultant | PCARD-REGISTERED NURSE |
| Consultant | Pediatric Cardiac ICU-REGISTERED NURSE |
| Consultant | Pediatric Cardiology-NURSE PRACTITIONER |
| Consultant | Pediatric Cardiology-REGISTERED NURSE |
| Consultant | Children's Hospital GI Lab-REGISTERED NURSE |
| Consultant | Allergy/Pulmonary-FELLOW |
| Consultant | Anesthesiology-FELLOW |
| Consultant | GI Medicine-FELLOW |
| Consultant | Medicine-Housestaff-FELLOW |
| Consultant | Neuro-Epilepsy-FELLOW |
| Consultant | Neuro-Pediatrics-FELLOW |
| Consultant | Pediatric Critical Care-FELLOW |
| Consultant | Pediatric Endocrinology-FELLOW |
| Consultant | Pediatric Gastroenterology-FELLOW |
| Consultant | Pediatric Hematology-FELLOW |
| Consultant | Pediatric Hospital Medicine-FELLOW |
| Consultant | Pediatric Infectious Disease-FELLOW |
| Consultant | Pediatric Pulmonary-FELLOW |
| Consultant | Pediatric Rheumatology-FELLOW |
| Consultant | Pediatric Surgery-FELLOW |
| Consultant | Cardiovascular Medicine-FELLOW |
| Consultant | Pediatric Cardiology-FELLOW |
| Consultant | Dentistry-PHYSICIAN |
| Consultant | Dermatology-PHYSICIAN |
| Consultant | Developmental Medicine-PHYSICIAN |
| Consultant | Emergency Medicine-PHYSICIAN |
| Consultant | Eye Institute-PHYSICIAN |
| Consultant | Hematology Oncology-PHYSICIAN |
| Consultant | Internal Medicine-PHYSICIAN |
| Consultant | Neuro Surgery-PHYSICIAN |
| Consultant | Ob-Gyn-PHYSICIAN |
| Consultant | Ortho-Pediatrics-PHYSICIAN |
| Consultant | Otolaryngology-PHYSICIAN |
| Consultant | Pediatric Critical Care-PHYSICIAN |
| Consultant | Pediatric Endocrinology-PHYSICIAN |
| Consultant | Pediatric Gastroenterology-PHYSICIAN |
| Consultant | Pediatric Genetics-PHYSICIAN |
| Consultant | Pediatric Hematology-PHYSICIAN |
| Consultant | Pediatric Hospital Medicine-PHYSICIAN |
| Consultant | Pediatric Infectious Disease-PHYSICIAN |
| Consultant | Pediatric Nephrology-PHYSICIAN |
| Consultant | Pediatric Neurology-PHYSICIAN |
| Consultant | Pediatric Pulmonary-PHYSICIAN |
| Consultant | Pediatric Rheumatology-PHYSICIAN |
| Consultant | Pediatric Surgery-PHYSICIAN |
| Consultant | Peds Emergency Medicine-PHYSICIAN |
| Consultant | Peds Outreach Medicine-PHYSICIAN |
| Consultant | Physical Medicine and Rehab-PHYSICIAN |
| Consultant | Plastics & Cosmetic Surgery-PHYSICIAN |
| Consultant | Radiation Oncology-PHYSICIAN |
| Consultant | Radiology-PHYSICIAN |
| Consultant | Urology-PHYSICIAN |
| Consultant | Vascular Surgery-PHYSICIAN |
| Consultant | Cardiovascular Medicine-PHYSICIAN |
| Consultant | Pediatric Cardiac Surgery-PHYSICIAN |
| Consultant | Pediatric Cardiology-PHYSICIAN |
| Consultant | Anesthesiology-RESIDENT PHYSICIAN |
| Consultant | Dermatology-RESIDENT PHYSICIAN |
| Consultant | Emergency Medicine-RESIDENT PHYSICIAN |
| Consultant | General Surgery-RESIDENT PHYSICIAN |
| Consultant | Internal Medicine-RESIDENT PHYSICIAN |
| Consultant | Kennedy Center-RESIDENT PHYSICIAN |
| Consultant | Med Peds-RESIDENT PHYSICIAN |
| Consultant | Medicine-Housestaff-RESIDENT PHYSICIAN |
| Consultant | Neuro Surgery-RESIDENT PHYSICIAN |
| Consultant | Neuro-Immunology-RESIDENT PHYSICIAN |
| Consultant | Neurology-RESIDENT PHYSICIAN |
| Consultant | Ob-Gyn-RESIDENT PHYSICIAN |
| Consultant | Ophthalmology-RESIDENT PHYSICIAN |
| Consultant | Oral Surgery-RESIDENT PHYSICIAN |
| Consultant | Ortho & Rehab-RESIDENT PHYSICIAN |
| Consultant | Otolrn Surg-RESIDENT PHYSICIAN |
| Consultant | Pediatric Hematology-RESIDENT PHYSICIAN |
| Consultant | Peds Emergency Medicine-RESIDENT PHYSICIAN |
| Consultant | Plastics & Cosmetic Surgery-RESIDENT PHYSICIAN |
| Consultant | Psychiatry-Housestaff-RESIDENT PHYSICIAN |
| Consultant | Radiology-RESIDENT PHYSICIAN |
| Consultant | Section Surgical Research-RESIDENT PHYSICIAN |
| Consultant | Thoracic Surgery-RESIDENT PHYSICIAN |
| Consultant | Urolog Surg-RESIDENT PHYSICIAN |
| Consultant | Pediatric Cardiology-RESIDENT PHYSICIAN |
| Consultant | Advanced Practice Clinics-MIDWIFE |
| Consultant | Children's Hospital Clinics-MEDICAL ASSISTANT |
| Consultant | Labor & Delivery-REGISTERED NURSE |
| Neonatal Fellow | Neonatology-FELLOW |
| Neonatal front line provider | Advanced Practice Clinics-NURSE PRACTITIONER |
| Neonatal front line provider | Advanced Practice Clinics-REGISTERED NURSE |
| Neonatal front line provider | Children's Hospital Nursing-NURSE PRACTITIONER |
| Neonatal front line provider | Hospital Nurse Practitioners-NURSE PRACTITIONER |
| Neonatal front line provider | In-Patient Nrs Practnrs-NURSE PRACTITIONER |
| Neonatal front line provider | General Pediatrics-PHYSICIAN |
| Neonatal front line provider | In-Patient Nrs Practnrs-PHYSICIAN ASSISTANT |
| Neonatal front line provider | Neonatology-RESIDENT PHYSICIAN |
| Neonatal front line provider | Neuro-Pediatrics-RESIDENT PHYSICIAN |
| Neonatal front line provider | Pediatrics-RESIDENT PHYSICIAN |
| Neonatal front line provider | Peds Outreach Medicine-RESIDENT PHYSICIAN |
| Neonatal nurse | Children's Hospital Admin-REGISTERED NURSE |
| Neonatal nurse | Children's Hospital Clinic Admin-REGISTERED NURSE |
| Neonatal nurse | Children's Hospital Clinics-LICENSED NURSE |
| Neonatal nurse | Children's Hospital Clinics-REGISTERED NURSE |
| Neonatal nurse | Children's Hospital Competencies-REGISTERED NURSE |
| Neonatal nurse | Children's Hospital Complex Care Competencies-REGISTERED NURSE |
| Neonatal nurse | Children's Hospital Executive Admin-REGISTERED NURSE |
| Neonatal nurse | Children's Hospital Float Pool-REGISTERED NURSE |
| Neonatal nurse | Children's Hospital Nursing-REGISTERED NURSE |
| Neonatal nurse | External Agency-REGISTERED NURSE |
| Neonatal nurse | General Pediatrics-REGISTERED NURSE |
| Neonatal nurse | In-Patient Nrs Practnrs-REGISTERED NURSE |
| Neonatal nurse | NICU-REGISTERED NURSE |
| Neonatal nurse | Neonatal Services-REGISTERED NURSE |
| Neonatal nurse | Neonatal/ Pediatric Transport-REGISTERED NURSE |
| Neonatal nurse | Neonatology-REGISTERED NURSE |
| Neonatal nurse | Nursing Admin-REGISTERED NURSE |
| Neonatal nurse | Pediatrics-REGISTERED NURSE |
| Neonatal nurse | School of Nursing-NON CREDENTIALED PROVIDER |
| Neonatologist | Neonatology-PHYSICIAN |
| Respiratory therapist | Neonatal/ Pediatric Transport-RESPIRATORY THERAPIST |
| Respiratory therapist | Peds Respiratory Care-RESPIRATORY THERAPIST |
| Support | Allied Health Non-Employee-SCRIBE |
| Support | Labor & Delivery-COORDINATOR |
| Support | Labor & Delivery-CLERK |
| Support | Admitting-COORDINATOR |
| Support | Admitting-MEDICAL ASSISTANT |
| Support | Central Registration-MEDICAL ASSISTANT |
| Support | Children's Hospital Holding Room/ PACU-COORDINATOR |
| Support | Children's Hospital Neuro & Neuro Surgery Competencies-COORDINATOR |
| Support | Children's Hospital Patient Safety-MENTAL HEALTH SPECIALIST |
| Support | Children's Hospital Performance Mgmt & Imp-REGISTERED NURSE |
| Support | HR TempForce-SPIRITUAL CARE |
| Support | Health Information Management-LICENSED NURSE |
| Support | Health Information Management-REGISTERED NURSE |
| Support | HealthIT Ambulatory Clinical-REGISTERED NURSE |
| Support | HealthIT ClinDoc-REGISTERED NURSE |
| Support | HealthIT Inpatient Orders-NURSE PRACTITIONER |
| Support | HealthIT Inpatient Orders-REGISTERED NURSE |
| Support | HealthIT Pharmacy-PHARMACIST |
| Support | Lifeflight-PARAMEDIC |
| Support | Lifeflight-REGISTERED NURSE |
| Support | Lifeflight-RESEARCH COORDINATOR - NON CLINICAL |
| Support | Medical Center IT CAST-REGISTERED NURSE |
| Support | NICU-COORDINATOR |
| Support | Nursing Informatics Services-REGISTERED NURSE |
| Support | Occup Therapy-OCCUPATIONAL THERAPIST |
| Support | Patient Financial Svcs-REGISTERED NURSE |
| Support | Pediatric Utilization Mgmt-UTILIZATION MANAGER |
| Support | Periop Support Svc(Scheduling)-REGISTERED NURSE |
| Support | Quality Safety Risk Prev-REGISTERED NURSE |
| Support | Risk and Insurance Management-REGISTERED NURSE |
| Support | STAM-COORDINATOR |
| Support | STAM-REGISTERED NURSE |
| Support | Transition Management Office-ANCILLARY STUDENT |
| Support | Transition Management Office-SOCIAL WORKER |
| Support | Transition Management Office-UTILIZATION MANAGER |
| Support | Transplant Financial-TRANSPLANT TEAM |
| Support | WIC-NURSE PRACTITIONER |
| Support | WIC-REGISTERED NURSE |
| Support | Care Connections-PATIENT ACCESS |
| Support | Center for Women's Health-PATIENT ACCESS |
| Support | Children's Hospital Access Services-PATIENT ACCESS |
| Support | Children's Hospital Access Services-REGISTERED NURSE |
| Support | Children's Hospital Clinics-PATIENT ACCESS |
| Support | Children's Hospital Competencies-PATIENT ACCESS |
| Support | Children's Hospital Complex Care Competencies-PATIENT ACCESS |
| Support | Children's Hospital GI-PATIENT ACCESS |
| Support | Children's Hospital Hem Onc Clinic-PATIENT ACCESS |
| Support | Children's Hospital Hemoc Competencies-PATIENT ACCESS |
| Support | Children's Hospital Neuro & Neuro Surgery Competencies-PATIENT ACCESS |
| Support | Children's Hospital Oto Audio Speech-PATIENT ACCESS |
| Support | Children's Hospital Radiology Access-PATIENT ACCESS |
| Support | Eye Institute-PATIENT ACCESS |
| Support | Home Care Services Admin-PATIENT ACCESS |
| Support | Ingram System for Timely Access-PATIENT ACCESS |
| Support | Internal Medicine-PATIENT ACCESS |
| Support | Murfreesboro Recovery-PATIENT ACCESS |
| Support | Neonatology-PATIENT ACCESS |
| Support | Neuroscience-PATIENT ACCESS |
| Support | Orthopaedic Institute Clinic-PATIENT ACCESS |
| Support | Ped Appt Line-PATIENT ACCESS |
| Support | Ped Gen Surgery-PATIENT ACCESS |
| Support | Ped Heart Institute-PATIENT ACCESS |
| Support | Ped Ortho-PATIENT ACCESS |
| Support | Ped Urology-PATIENT ACCESS |
| Support | Peds ED Registration-PATIENT ACCESS |
| Support | Pre-Visit Collections-PATIENT ACCESS |
| Support | Pediatric Cardiac Surgery-PATIENT ACCESS |
| Support | Pediatric Cardiology-PATIENT ACCESS |
| Support | HealthIT Lab System-PHLEBOTOMIST |
| Support | Biomedical Informatics-PHYSICIAN |
| Support | Occ Health-PHYSICIAN |
| Support | Central Scheduling-CLERK |
| Support | Children's Hospital Administration-CLERK |
| Support | Children's Hospital Nutrition Support Srvcs-CLERK |
| Support | Medical Record Operations-CLERK |
| Support | NICU-CLERK |
| Support | Obstetrics-CLERK |
| Support | Orthopaedics-CLERK |
| Support | Pediatric Emergency -CLERK |
| Support | Pediatric Hematology/Oncology-CLERK |
| Support | Pediatric Medicine Acute Care -CLERK |
| Support | Peds ED Registration-CLERK |
| Support | Vascular Institute Non-Inv Diag Support-CLERK |
| Support | Vascular Institute OP Clinic-CLERK |
| Support | Children's Hospital Radiology Access-CLERK |
| Support | PCARD-CLERK |
