# Supplementary material for: Measuring Collaboration Through Concurrent Electronic Health Record Usage: Network Analysis Study
Source: JMIR Med Inform. 2021 Sep 3;9(9):e28998. doi: 10.2196/28998 (PMC8449299; doi:10.2196/28998)
Supplement: Multimedia Appendix 3 [file medinform_v9i9e28998_app3.docx]

**Multimedia Appendix 3.** Survey questions.

The respondents to our survey were targeted and not randomly selected. The decision for targeting was so that our inferred collaboration relationships could be interpreted by experts who are knowledgeable on interactions of health workers in the NICU.

The 12 survey questions are shown in Table S3. Each expert in this study was provided with the same survey, such that all questions were presented in the same order. The survey aims to assess virtual interactions occurring in electronic health record systems. An example of an interaction between healthcare workers in eStar: a healthcare worker entered an order, and another healthcare worker read the order and modified it.

In this study, healthcare workers are from the following eight roles:

(1) Neonatal front line provider (e.g., nurse practitioner, physician assistant, hospitalist, or resident physician)

(2) Neonatologist

(3) Consultant (e.g., surgery physicians, OB/GYN physician, hematology physician, radiology physician, anesthesiologist, genetics counselor)

(4) Ancillary staff (e.g., registered dietitian, social worker, case manager, technician, phlebotomist)

(5) Neonatal fellow

(6) Neonatal nurse

(7) Respiratory therapists

(8) Support staff (e.g., clerk, IT staff, coordinator, medical assistant, financial and administrative, supply chain)

**Table S1.** The twelve survey questions. Each question corresponds to a collaborative relationship between two healthcare workers. We randomly selected six relationships with high strength learned from our approaches and six with low. The respondents did not know the learned strengths of the relationships when they completed the surveys. eStar is the name of the electronic health record system used at Vanderbilt University Medical Center.

| **Question 1:** To what extent do you believe **a neonatal front line provider**interacts with**a consultant**in eStar to manage a NICU patient?  Examples of **a front line provider**: nurse practitioner, physician assistant, hospitalist, or resident physician.  Examples of a**consultant**: surgery physicians, OB/GYN physician, hematology physician, radiology physician, anesthesiologist, genetics counselor. |
| --- |
| ☐Not at all likely  ☐Slightly likely  ☐Moderately likely  ☐Very likely  ☐Completely likely |
| **Question2:**To what extent do you believe**a neonatal front line provider**interacts with**a neonatologist**in eStar to manage a NICU patient?  Examples of **a front line provider:** nurse practitioner, physician assistant, hospitalist, or resident physician. |
| ☐Not at all likely  ☐Slightly likely  ☐Moderately likely  ☐Very likely  ☐Completely likely |
| **Question 3:**To what extent do you believe **an ancillary staff**interacts with**a support staff**in eStar to manage a NICU patient?  Examples of **an ancillary staff**: registered dietitian, social worker, case manager, technician, phlebotomist.  Examples of **a support staff**: clerk, IT staff, coordinator, medical assistant, financial and administrative, supply chain. |
| ☐Not at all likely  ☐Slightly likely  ☐Moderately likely  ☐Very likely  ☐Completely likely |
| **Question 4:**To what extent do you believe**an ancillary staff**interacts with**a consultant**in eStar to manage a NICU patient?  Examples of **an ancillary staff** : registered dietitian, social worker, case manager, technician, phlebotomist.  Examples of **a consultant**: surgery physicians, OB/GYN physician, hematology physician, radiology physician, anesthesiologist, genetics counselor. |
| ☐Not at all likely  ☐Slightly likely  ☐Moderately likely  ☐Very likely  ☐Completely likely |
| **Question 5:** To what extent do you believe**a neonatal nurse**interacts with **a neonatal fellow**in eStar to manage a NICU patient? |
| ☐Not at all likely  ☐Slightly likely  ☐Moderately likely  ☐Very likely  ☐Completely likely |
| **Question 6:**To what extent do you believe**a neonatal nurse** interacts with**a front line provider**in eStar to manage a NICU patient?  Examples of **a front line provider**: nurse practitioner, physician assistant, hospitalist, or resident physician. |
| ☐Not at all likely  ☐Slightly likely  ☐Moderately likely  ☐Very likely  ☐Completely likely |
| **Question 7:** To what extent do you believe**a neonatal front line provider**interacts with **a neonatal fellow**in eStar to manage a NICU patient?  Examples of **a front line provider**: nurse practitioner, physician assistant, hospitalist, or resident physician. |
| ☐Not at all likely  ☐Slightly likely  ☐Moderately likely  ☐Very likely  ☐Completely likely |
| **Question 8:**To what extent do you believe **a neonatal front line provider**interacts with**an ancillary staff**in eStar to manage a NICU patient?  Examples of**a front line provider**: nurse practitioner, physician assistant, hospitalist, or resident physician.  Examples of **an ancillary staff**: registered dietitian, social worker, case manager, technician, phlebotomist. |
| ☐Not at all likely  ☐Slightly likely  ☐Moderately likely  ☐Very likely  ☐Completely likely |
| **Question 9:**To what extent do you believe **an ancillary staff**interacts with**a neonatal nurse**in eStar to manage a NICU patient?  Examples of **an ancillary staff**: registered dietitian, social worker, case manager, technician, phlebotomist. |
| ☐Not at all likely  ☐Slightly likely  ☐Moderately likely  ☐Very likely  ☐Completely likely |
| **Question 10:**To what extent do you believe**an ancillary staff**interacts with**a neonatal fellow**in eStar to manage a NICU patient?  Examples of **an ancillary staff:** registered dietitian, social worker, case manager, technician, phlebotomist. |
| ☐Not at all likely  ☐Slightly likely  ☐Moderately likely  ☐Very likely  ☐Completely likely |
| **Question 11:** To what extent do you believe**a support staff**interacts with **a neonatal nurse**in eStar to manage a NICU patient?  Examples of **a support staff** : clerk, IT staff, coordinator, medical assistant, financial and administrative, supply chain. |
| ☐Not at all likely  ☐Slightly likely  ☐Moderately likely  ☐Very likely  ☐Completely likely |
| **Question 12:**To what extent do you believe**a support staff**interacts with**a neonatal fellow** in eStar to manage a NICU patient?  Examples of **a support staff** : clerk, IT staff, coordinator, medical assistant, financial and administrative, supply chain. |
| ☐Not at all likely  ☐Slightly likely  ☐Moderately likely  ☐Very likely  ☐Completely likely |
