# Supplementary material for: Measuring Collaboration Through Concurrent Electronic Health Record Usage: Network Analysis Study
Source: JMIR Med Inform. 2021 Sep 3;9(9):e28998. doi: 10.2196/28998 (PMC8449299; doi:10.2196/28998)
Supplement: Multimedia Appendix 4 [file medinform_v9i9e28998_app4.docx]

**Multimedia Appendix 4.** Summary statistics of neonatal intensive care unit experts participating in online surveys.

**Table S1.** NICU experts of the VUMC who participated in the survey. The average number of years working at the NICU is 5.65.

| **Number of Respondents** | **Profession** | **Average Number of Years** |
| --- | --- | --- |
| 4 | Neonatologist | 10.00 |
| 3 | Neonatal fellow | 2.33 |
| 3 | Neonatal nurse | 3.58 |
| 2 | Nurse practitioner | 8.00 |
| 1 | Respiratory therapist | 4 |
