# Supplementary material for: Measuring Collaboration Through Concurrent Electronic Health Record Usage: Network Analysis Study
Source: JMIR Med Inform. 2021 Sep 3;9(9):e28998. doi: 10.2196/28998 (PMC8449299; doi:10.2196/28998)
Supplement: Multimedia Appendix 5 [file medinform_v9i9e28998_app5.docx]

**Multimedia Appendix 5.** Likert scores of 12 collaboration relationships surveyed from neonatal intensive care unit experts, and the plot of standardized residuals for the linear regression model.


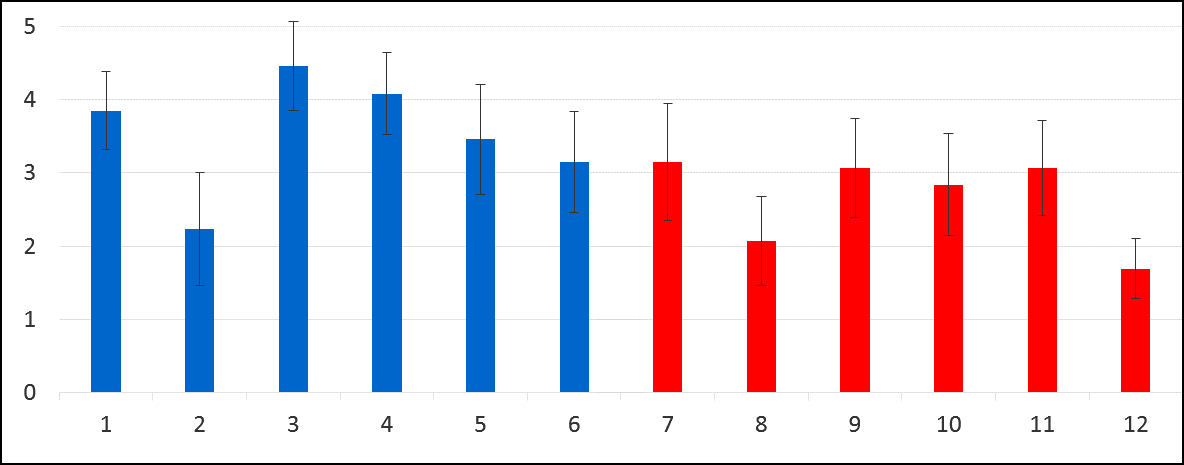


**Figure S1** Average Likert scores for the 12 collaboration relationships. The bar graphs also show the 95% confidence intervals. The blues are collaboration relationships with high likelihoods, and the reds are low likelihoods.


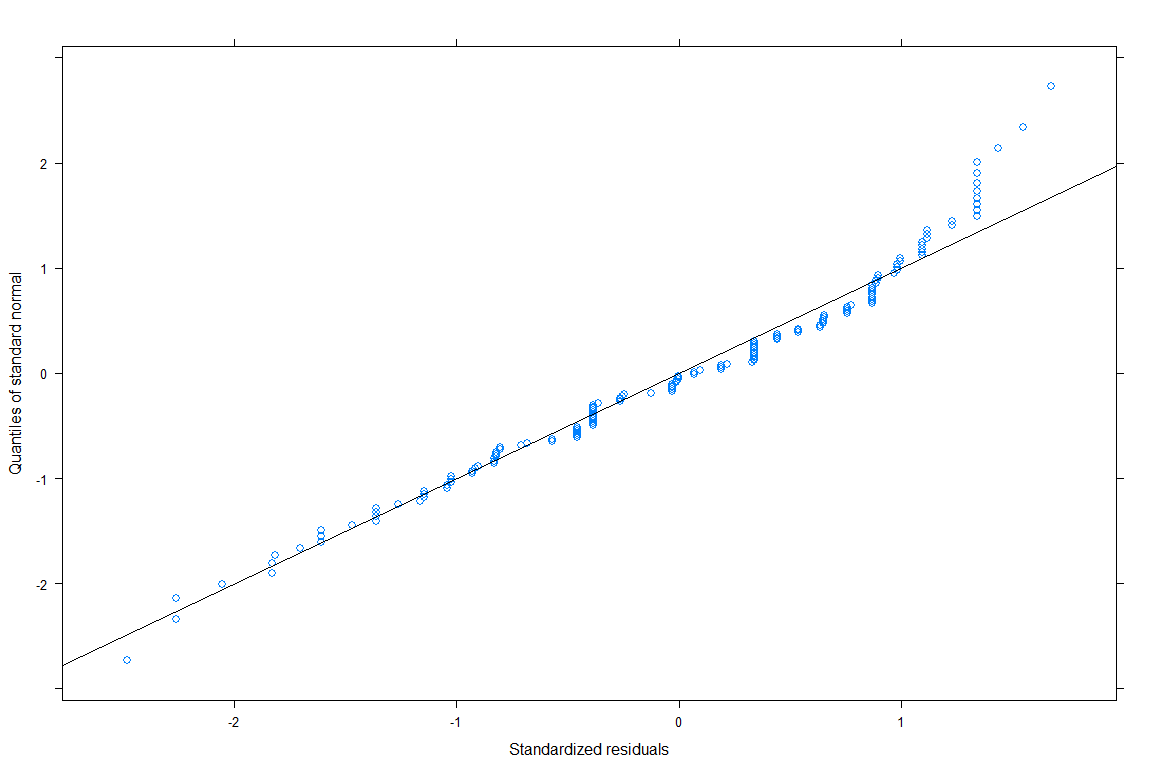


**Figure S2** The plot of standardized residuals for the linear regression model in our validation study.
