# Supplementary material for: Measuring Collaboration Through Concurrent Electronic Health Record Usage: Network Analysis Study
Source: JMIR Med Inform. 2021 Sep 3;9(9):e28998. doi: 10.2196/28998 (PMC8449299; doi:10.2196/28998)
Supplement: Multimedia Appendix 6 [file medinform_v9i9e28998_app6.docx]

**Multimedia Appendix 6.** Determination of the number of clusters, and visualization of the cluster-cluster relationships.

**
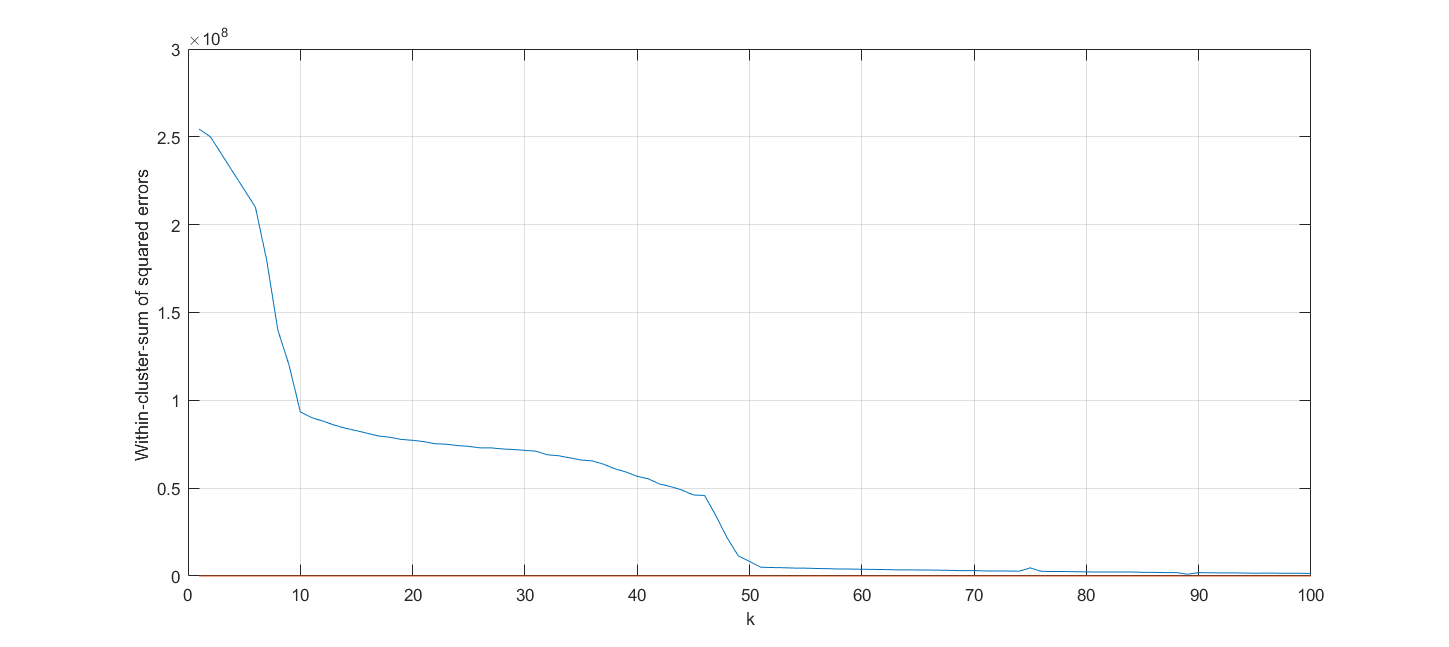
**

**Figure S1** The plot of the total within-cluster-sum of squared errors as a function of the number of clusters k.


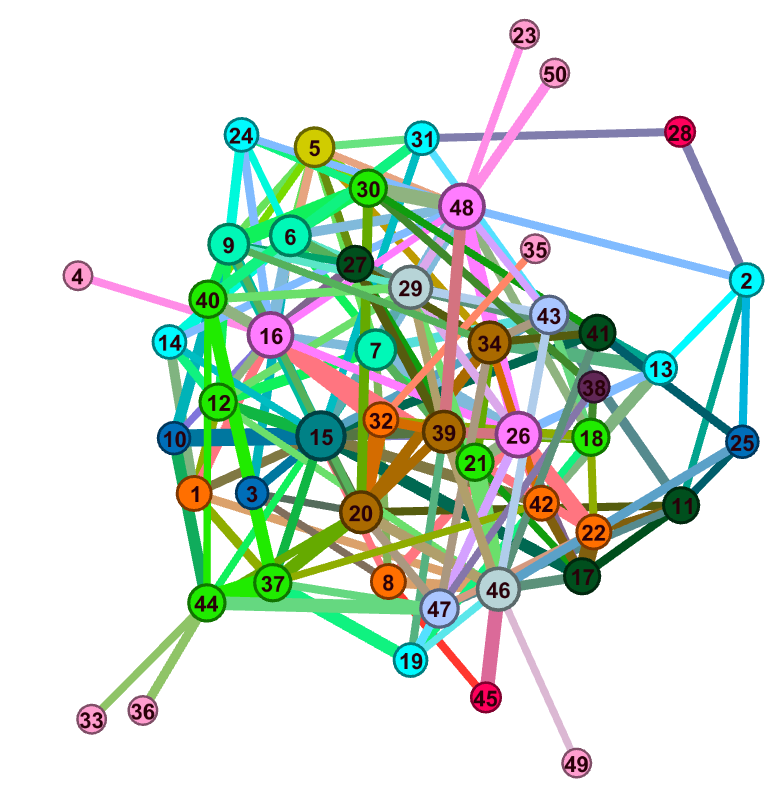


**Figure S2** A visualization of the cluster-cluster relationships from **Figure 7**. Each node is a cluster of collaborative intervals, and each edge indicates that there are Intervals from both nodes that share the same collaborative sessions. The weight of the edge indicates the strength of the connection between the two nodes, or in other words, the number of intervals that share the same collaborative session. The weight is indicated by its width. The number of directed connections determines the node size. Nodes with the same color have the same number of connections.
